# It Takes a Village: The Multifaceted Immune Response to *Mycobacterium tuberculosis* Infection and Vaccine-Induced Immunity

**DOI:** 10.3389/fimmu.2022.840225

**Published:** 2022-03-10

**Authors:** Sasha E. Larsen, Brittany D. Williams, Maham Rais, Rhea N. Coler, Susan L. Baldwin

**Affiliations:** ^1^ Center for Global Infectious Disease Research, Seattle Children's Research Institute, Seattle Children's Hospital, Seattle, WA, United States; ^2^ Department of Global Health, University of Washington, Seattle, WA, United States; ^3^ Department of Pediatrics, University of Washington School of Medicine, Seattle, WA, United States

**Keywords:** infection, immunity, vaccines, BCG, *Mycobacterium tuberculosis*, prevention of infection (POI), prevention of disease (POD)

## Abstract

Despite co-evolving with humans for centuries and being intensely studied for decades, the immune correlates of protection against *Mycobacterium tuberculosis* (Mtb) have yet to be fully defined. This lapse in understanding is a major lag in the pipeline for evaluating and advancing efficacious vaccine candidates. While CD4+ T helper 1 (TH1) pro-inflammatory responses have a significant role in controlling Mtb infection, the historically narrow focus on this cell population may have eclipsed the characterization of other requisite arms of the immune system. Over the last decade, the tuberculosis (TB) research community has intentionally and intensely increased the breadth of investigation of other immune players. Here, we review mechanistic preclinical studies as well as clinical anecdotes that suggest the degree to which different cell types, such as NK cells, CD8+ T cells, γ δ T cells, and B cells, influence infection or disease prevention. Additionally, we categorically outline the observed role each major cell type plays in vaccine-induced immunity, including *Mycobacterium bovis* bacillus Calmette-Guérin (BCG). Novel vaccine candidates advancing through either the preclinical or clinical pipeline leverage different platforms (e.g., protein + adjuvant, vector-based, nucleic acid-based) to purposefully elicit complex immune responses, and we review those design rationales and results to date. The better we as a community understand the essential composition, magnitude, timing, and trafficking of immune responses against Mtb, the closer we are to reducing the severe disease burden and toll on human health inflicted by TB globally.

## Introduction

Pulmonary tuberculosis (TB) caused by *Mycobacterium tuberculosis* (Mtb) was the leading infectious killer globally for the 4 years (1) predating the severe acute respiratory syndrome corona virus 2 (SARS-CoV-2) pandemic (2). Approximately 1.5 million individuals succumbed to TB in 2020, up from 1.4 million in 2019, marking the first increase in global TB deaths in more than a decade (3, 4). Furthermore, the World Health Organization (WHO) and others estimate that COVID-19-related disruptions in care, including a 21% reduction in people receiving care for TB in 2020, could result in an additional half million deaths (5), likely in low- and middle-income countries (LMICs), which continue to bear a disproportionate burden of TB (6, 7). New therapies or interventions aimed towards prevention of infection (POI), prevention of disease (POD), and subsequent transmission are urgently needed (8). Moreover, Mtb drug resistance (DR) is steadily increasing globally. In each year since 2017, roughly half a million Mtb-infected individuals developed rifampicin resistance and ~80% of those cases had multidrug-resistant (MDR) TB (1, 3, 9). Focused efforts to design and evaluate low-cost and highly effective TB vaccines are urgently needed as current interventions alone are insufficient by many models (10, 11) to achieve the WHO End TB Strategy milestones. Several TB vaccines have been tested in clinical trials; however, only *Mycobacterium bovis* Bacillus Calmette Guérin (BCG) (0%–80% efficacy) (12) and M72/AS01_E_ (~50% effective against the progression of TB disease) (13, 14) have shown protection in humans. Additionally, no defined correlates of protection (COP) are solidified for Mtb infection or TB disease. Therefore, it is worth taking note of the immune responses elicited by these vaccines (15) and others showing promise in the pipeline. The aim of this review is to focus on immune cells and immunological mediators against Mtb, induced both preclinically and clinically by TB vaccine candidates and BCG, that have been overshadowed by a myopic focus on CD4+ T helper 1 (TH1) cells. We hope that this collection informs future immune efficacy endpoints and helps draw the field closer to predictive COP endpoints.

The TB vaccine landscape is poised to make formidable leaps forward. This is in part due to courageous work by the international research communities and seminal publications demonstrating near sterilizing protection from Mtb challenge in preclinical models (16). Findings of a recent study showing that intravenous (i.v.) BCG prevents or substantially limits Mtb infection in a susceptible rhesus macaque model (16) provides a benchmark against which future vaccines will be evaluated and importantly a new framework to understand the immune correlates and mechanisms of protection against TB. For example, many arms of the immune response are engaged following i.v. BCG delivery compared to intradermal (i.d.) delivery. In the airway (bronchioalveolar lavage fluid), early γδ T-cell, invariant natural killer T cell (iNKT), natural killer (NK) cell, B-cell, neutrophil, myeloid dendritic cell (mDC), and mucosal-associated invariant T (MAIT) cell responses are observed. In the i.v. group, both memory CD4+ and CD8+ T cells producing TH1 and TH17 cytokines were captured, whereas only CD4+ responses are elicited in the i.d. cohort (16). Mucosal airway and peripheral antibody responses (IgG, IgA, and IgM) were also highest in the i.v. group, 4 weeks after vaccination (16, 17). These data help to highlight the diversity of immune responses that may be working in concert to afford protection from Mtb, but to date have not been so well captured across a single study with robust correlating efficacy. Between this seminal investigation and other recent groundbreaking discoveries of immune COP, the research community is highlighting the critical roles of commonly overlooked and bypassed immune responses.

## Cell Subsets, Widening the View

**Figure 1:**
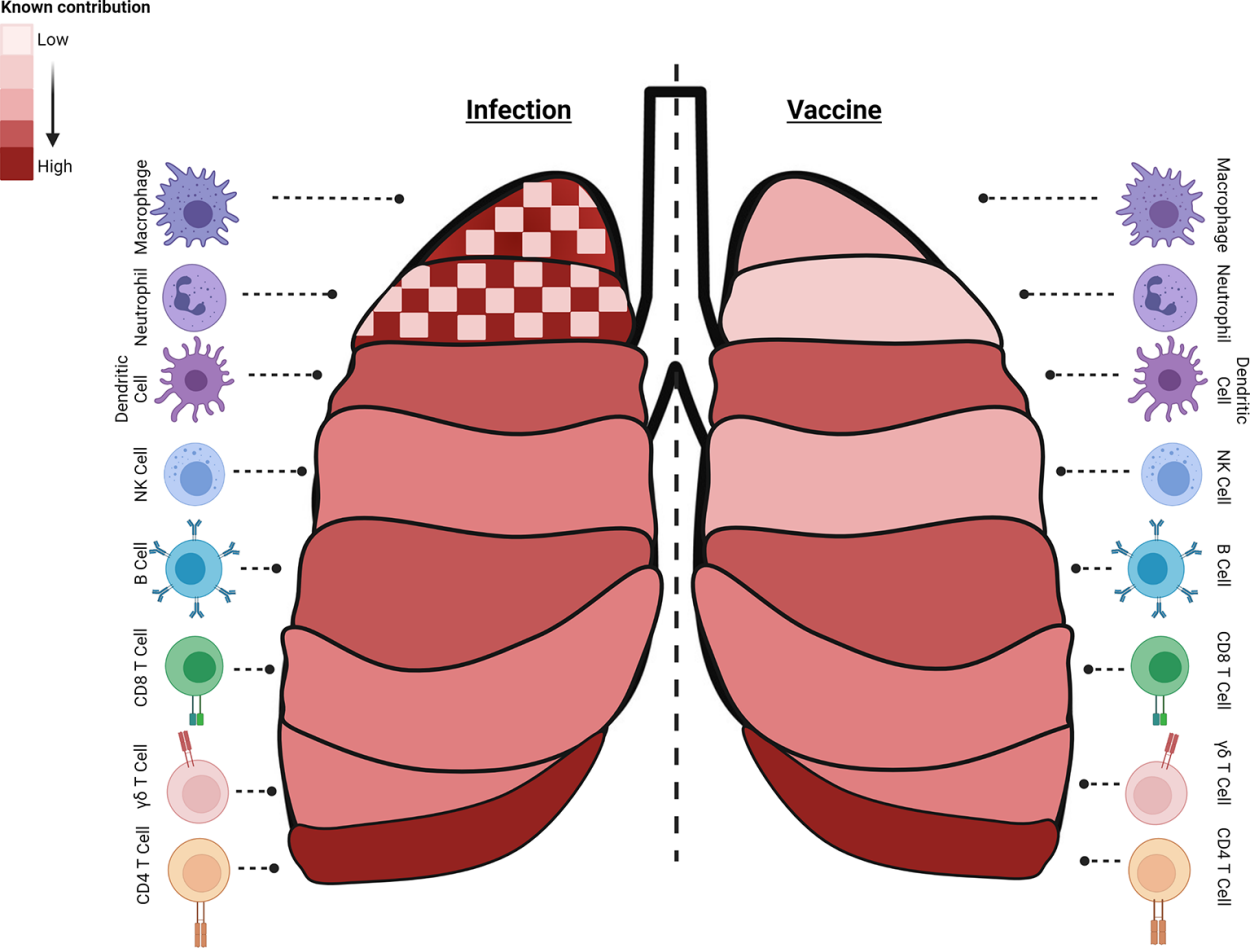
Immune cell subsets with critical roles during different stages of Mtb infection or generated by specific vaccine strategies. Known contributions (heatmap of light: low, dark: high, checked: mixed) of specific subsets are outlined during infection (left) or following vaccine induction (right). Macrophages, neutrophils, and B cells have known and defined contributions to controlling infection and preventing disease. Interestingly, both macrophages and neutrophils can contribute to Mtb control or serve as a niche for bacterial growth and dissemination and their dual role is highlighted (checked). Dendritic cells participate in early stages of infection and post vaccination as an APC. NK cells contribute moderately to infection control and little is known about their induction with different vaccination regimens. Antigen-specific cytolytic CD8+ T target Mtb and Mtb-infected cells, and their part in controlling latent infection, important in POD vaccination strategies, is expanding. While a multi-faceted immune response is induced to control Mtb infection, vaccine-induced immune responses essential for protection by many of these cell subsets are still understudied as endpoints. The summary presented here is a collection of information from many models and clinical data and do not reflect the data known or observed for each model (created with BioRender.com).

While most primary endpoints for vaccine immunogenicity preferentially evaluate anti-Mtb specific TH1-type responses (18), the full mechanism of protection has yet to be determined (19). Despite many candidates inducing robust classical TH1 CD4+ T-cell responses in preclinical and clinical trials, no candidate has met the target product profile for an efficacious TB vaccine, so we need to collectively look beyond this subset. In a phase 2b safety and efficacy trial of candidate MVA85A, researchers observed robust TH1 responses but a dramatic lack of efficacy in previously BCG-vaccinated neonates (20). This is a recent example of how the reliance on this primary endpoint has led to disappointing efficacy results and stalling of funding support for clinical candidates. Indeed, protection from Mtb infection and TB disease is likely a multifaceted process involving many cell types beyond canonical CD4+ T cells and their main proinflammatory cytokine interferon gamma (IFNγ). In a 2017 review of polyfunctional CD4+ T cells induced by BCG and TB vaccine candidates in preclinical and clinical studies, Lewinsohn and colleagues conclude that this subset is likely not sufficient for protective efficacy (18) and suggested that further studies were warranted to specifically address their mechanistic role in protection. Now, several years later, in the collaborative cross mouse model (21), researchers have succinctly uncoupled the magnitude of IFNγ expression and subsequent control of Mtb, where a proportion of genotypes evaluated for Mtb susceptibility had low IFNγ production but still controlled the infection (22). This dissociation found in mice has also been recently observed in human resister (RSTR) cohorts. A prospective household contact study in Uganda found that RSTRs consistently test negative by tuberculin skin testing (TST) despite constant Mtb challenge, suggesting IFNγ-independent immunity in this population (23). Given these findings, it is even more important to diversify our understanding of the cellular contributors to Mtb immunity. Here, we have reviewed the effector functions, role(s) during infection, and what is known about specific cell subsets’ [alveolar macrophages (AM), neutrophils, DCs, NK cells, B cells, CD8+ T cells, and γδ T cells] induction with different vaccine candidates in the preclinical or clinical pipeline (**Figure 1** and **Table 1**). While animal models of TB do not fully represent the spectrum of human infection and disease pathogenesis or align within the preclinical pipeline itself, they are improving and can be informative (95); therefore, the information presented here reflects a review of primary *in vitro* or *ex vivo* data from animal models as well as observations from human clinical studies.

**Table 1:** Clinical vaccine candidate status and induction of cell subsets.

Candidate*	Route	Interval	Stage	POD or POI	Evidence cell subset is vaccine-induced							
					CD4^+^ T	CD8^+^ T	γδ T	B cell	NK	DC	Mac	PMN
**MTBVAC** (24–27)	i.d.	1 ×	Ph 2a	POI	YpYc	YpYc	Yp	YpYc	Yp	(-)	(-)	(-)
**VPM1002** (28–31)	i.d.	1 ×	Ph 3	POI & POD	YpYc	YpYc	Yp	YpYc	Yp	(-)	(-)	Yp
**Ad5 Ag85A** (32–34)	i.m. or aero	1 ×	Ph 1	POI	YpYc	YpYc	(-)	(-)	(-)	(-)	(-)	(-)
**ChadOx1.85A + MVA** (35–37)	i.m.	Prime—8 weeks boost	Ph 1	POI	YpYc	YpYc	(-)	YpYc	(-)	(-)	(-)	(-)
AEC/BC02** (38)	i.m.	6 × 2 weeks	Ph 1b	POI	(-)	(-)	(-)	(-)	(-)	(-)	(-)	(-)
TB/Flu04L*** (39)	i.n.	2 × 3 weeks	Ph 2a	POI & POD	Yp	(-)	(-)	(-)	(-)	(-)	(-)	(-)
**ID93+GLA-SE** (40–53)	i.m.	3 × 4 weeks; or 2 × 8 weeks, or 3 × D0, weeks 4 and 16	Ph 2a	POI & POD	YpYc	Yp	(-)	YpYc	Yc	YpYc	Yp	Yp
**GamTBVac** (54–56)	s.c.	2 × 8 weeks	Ph 2a	POI	YpYc	(-)	(-)	YpYc	(-)	Yp	(-)	(-)
**M72+ASO1** (13, 14, 53, 57–65)	i.m.	2 × 4 weeks	Ph 2b	POI & POD	YpYc	Yc	(-)	Yc	Yc	Yp	Yp	Yp
**DAR-901** (66–68)	i.d.	3 × 8 weeks	Ph 2b	POI	Yc	(-)	(-)	YpYc	(-)	(-)	(-)	(-)
**H56:IC31** (53, 69–79)	i.m.	2 or 3 × 8 weeks	Ph 2b	POI & POD	YpYc	(-)	(-)	YpYc	(-)	Yp^^^Yc	Yp^^^	Yp^^^
**BCG revaccination** (53, 74, 80–84)	i.d.	1 ×	Ph 2b	POI	Y_C_	Y_C_	Y_C_	YpYc	Y_C_	(-)	(-)	(-)
**MIP** (85–91)	i.d.	6 × 2 weeks	Ph 3	POI	YpYc	YpYc	(-)	Yp	(-)	(-)	Yp	Yp
**RUTI** (92–94)	i.m.	1 ×	Ph 2a	POD	YpYc	YpYc	(-)	(-)	(-)	(-)	Yp	(-)

*Candidates in Phase 1 clinical trials or beyond included, based on the Tuberculosis Vaccine Initiative Pipeline Tracker (www.treatmentactiongroup.org) in 2021. ELISPOT or T-cell proliferative assay data were omitted if they did not specify the subset tested.

**Phase 1 clinical trial for AEC/BC02 was completed in 2019 but as yet the results have not been reported.

***Phase 1 clinical trial for TB/Flu04L was completed in 2015 but as yet the results have not been reported.

POI, prevention of infection, pre-infection target population; POD, prevention of disease, post-infection target population.

Y_p_ = yes, observed in preclinical studies; T_c_ = yes, observed in clinical studies; (-) not yet observed or reported for candidate.

i.d., intradermal; i.m, intramuscular; i.n., intranasal; aero, aerosol; s.c., subcutaneous.

^^^H56 with CAF01 i.m. prime/mucosal pull or with IC31 activation alone.

### Neutrophils/PMNs

*Effector functions.* Innate immune cells such as neutrophils have been shown to help mediate early inflammatory responses that are critical for controlling infection (96–98). Initial immunology work focused on the role of specific phagocytes in infection, leaving neutrophils understudied and underappreciated. However, neutrophils account for 50%–80% of all circulating white blood cells in humans and contribute to the innate immune response *via* phagocytosis of invading bacteria, degranulation, and subsequent secretion of cytokines such as tumor-necrosis factor-alpha (TNF-α) and interleukin 1 (IL-1) (96, 99–102). Neutrophils provide a non-specific immediate innate response that helps contain and control invading pathogens, preventing dissemination and recruiting other cell types. Discovery of the various functions of neutrophils such as neutrophil extracellular trap (NET) formation, phagocytosis, heterogeneity, and plasticity has increased and opened new avenues in recent neutrophil research (103). For example, NET structures, made up of sticky extruded DNA, are unique to neutrophils and not only limit microbial spread and dissemination, but also enhance the concentration of microbicidal agents in human *ex vivo* evaluations (104–107). Neutrophils secrete reactive oxygen species (ROS), elastase, collagenase, and myeloperoxidase, factors that can both combat invading mycobacteria and, when overabundant, damage host cells in a nonselective manner (98, 102, 108).

**Figure 2:**
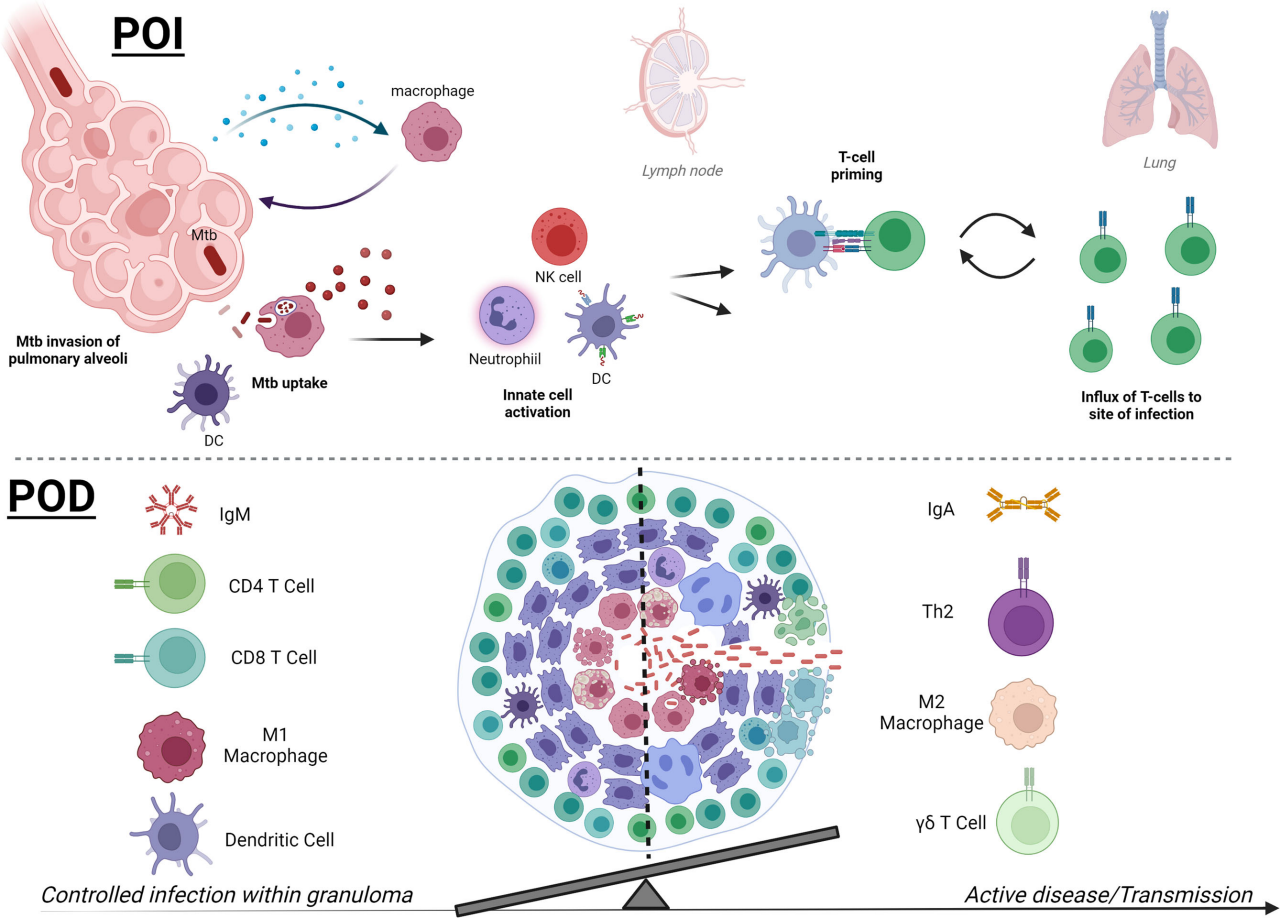
Mtb therapies focus on two main strategies, early prevention of infection or later prevention of disease. In early infection (POI, upper), detection of Mtb bacilli in the pulmonary alveoli by macrophages leads to downstream activation of innate immune cells, which may include neutrophils, NK cells, and DCs. Activated APCs can then prime T cells (CD4+ or CD8+) for further Mtb-specific adaptive responses to target Mtb and Mtb-infected cells and control infection. Novel therapies are working to skew innate immune responses to be protective and less permissive and accelerate T-cell priming and effector responses that traffic to the pulmonary space. Later stages of Mtb infection (POD, lower) and pathology are defined by formation of a granuloma, which contains Mtb by a surrounding composition of immune cells. While a hallmark of disease, there are still questions surrounding the factors that can affect granuloma formation and composition that can help resolve infection and prevent progression to active disease. Containment and POD progression seem to correlate with IgM, robust pulmonary CD4+ and CD8+ T cells, and activated inflammatory M1 macrophages and DCs at the granuloma. In contrast, regulatory TH2 CD4+ T cells, abundance of IgA or IgG4, and M2 macrophages are more associated with loss of control. Higher peripheral γδ T cells are associated with active TB disease in humans, but their direct role in granuloma control or bacterial dissemination requires further study. The balance of both the composition and magnitude of specific cellular and humoral players is becoming clearer and POD vaccine strategies will be able to benchmark these parameters in preclinical and clinical studies. The summary presented here is a collection of information from many models and clinical data and does not reflect the data known or observed for each model (created with BioRender.com).

Neutrophils in circulation can be recruited to lung parenchyma by cytokines, leading to their activation and phagocytosis of pathogen. In preclinical rodent models, CXC chemokines are a potent chemoattractant pulling neutrophils from circulation into an infected or damaged lung space (109). Neutrophils are also an important amplifying cell as they are a significant source of specific cytokines that help promote early recruitment and activation of other innate immune cells, contributing to cellular immunity against mycobacterial infection, as observed in a Balb/c mouse model challenged with H37Rv Mtb (110) (**Figure 2**). For example, *ex vivo* human neutrophils have been shown to modulate the effector mechanisms of resident AM (111), another crucial cell for control of Mtb, through the localization of antimicrobials and proteins like heat shock protein 72 (Hsp72), which induce inflammatory responses in AMs. Neutrophils employ an arsenal of bactericidal proteolytic enzymes that are in an arms race with evasive countermeasures deployed by certain bacterial processes (112). Therefore, neutrophil enzyme activation and secretion help shape the early innate immune response during bacterial infection (111).

*Role in Mtb infections.* While neutrophils provide early protection and participate in the early formation of granuloma structures, they can also be detrimental to the host if overabundant in later TB disease states. Studies of human neutrophils *ex vivo* observe that this subset can directly kill virulent Mtb lineages and suggest that TNF stimulation can aid this killing and the mechanism may be nonoxidative (113, 114). Indeed, neutrophils from patients with chronic granulomatous disease, which harbor defects in certain oxidase pathways, are equally efficient at Mtb killing compared to normal healthy control cells (113). However, there are many mechanisms by which neutrophils kill Mtb and eliminating one pathway may be compensated for by another. For example, human neutrophils have been shown to release lysosomal enzymes, human neutrophil peptides, and ROS, which can directly lyse Mtb in *ex vivo* co-culture situations (115, 116). Furthermore, depletion of neutrophils from patient whole blood samples significantly reduces the ability of those samples to restrict mycobacterial growth of both recombinant BCG and Mtb containing luciferase for kinetic evaluations (116). Arguably, neutrophils’ most iconic tool against bacterial pathogens are NETs, which play a significant role in controlling Mtb and subsequent immune activation. Indeed, DNA NET induction has been shown to be induced by Mtb in a guinea pig model (117), be at higher levels in plasma in patients with active TB disease (118), and activate human macrophages *ex vivo (* 105). Mtb-induced NETs can also help sequester toxic contents from dying neutrophils, including lysozyme, ion chelators (calgranulin), and histones (119), to limit damage to surrounding tissue, which have been observed in humans and preclinical models (105, 106). NETs play a vital role in the partnership between neutrophils and macrophages. As mentioned above, Hsp72-containing NETs trigger a pro-inflammatory response in resident AMs, inducing the release of IL-6, TNF-α, and IL-1β (105). Therefore, these important innate immune cells can help contain the infection and contribute to early granuloma formation (**Figure 2**) (104–107, 113, 114). Despite these many tools for Mtb killing and growth inhibition, there is also evidence of bacterial immune evasion from neutrophils. For instance, neutrophils isolated from healthy human blood have been shown to effectively phagocytose Mtb *ex vivo* but not kill up to 6 h in co-culture, eluding bacterial escape mechanisms (120). In these studies, neutrophils were activated but also underwent necrosis, which may enable Mtb survival (120).

An effective host innate immune response against Mtb is based not only on successful cell-mediated killing of *Mtb*, but also on the efficient regulation of innate immune cells, often *via* cytokines (114, 121). Activated neutrophils express TNF-α, IL-8, and granzyme effectors, which all further influence other cell subsets. TNF-α stimulates dendritic cells and macrophage differentiation and activation, which are vital host responses to Mtb infection (100, 101, 122). Additionally, TNF-α aids in the activation of T cells and helps promote granuloma formation (123, 124). In response to Mtb, neutrophils have also been shown to secrete the chemokine IL-8 (125), which has autocrine properties, enhancing direct neutrophil killing of Mtb, as well as paracrine recruitment of leukocytes to areas of granuloma formation (126–130). Interestingly, IL-8 concentrations are increased following the completion of a 9-month-long antibacterial therapy of Mtb both *in vivo* and in LPS-stimulated *ex vivo* plasma. Lastly, neutrophils in macaque and human granulomas have been shown to be significant contributors of granzyme B (GRZB) (131) and that GRZB+ expression in neutrophils positively associated with higher granuloma bacterial loads. GRZB is known to act on intracellular substrates including pro-caspase 3, contributing to pathogen clearance (132), but in the study discussed above, it correlates with a negative outcome (131). These data collectively suggest that based on timing and location, responses from neutrophils can be beneficial or detrimental and that intermediates like cytokines and chemokines play complex roles in these processes (131–134).

The role of neutrophils in TB disease progression is of importance. Whereas early neutrophil responses may be beneficial for reasons outlined above, several inflammatory mediators stemming from neutrophils cause lung damage and contribute to the development of caseous necrotic granulomas leading to active TB disease and transmission of Mtb (**Figure 1**). An important contribution to understanding the role of neutrophils in the late stage of active TB disease was the human blood IFN signature of active disease derived from neutrophils (135). In addition, a recent review discusses neutrophil-specific mediators that impact destructive inflammation resulting in increased disease and mortality (136). Indeed, disease severity and reduction of long-term pulmonary function seem to correlate with neutrophil mechanisms in patients with TB disease (136). These mediators include matrix metalloproteinases (MMPs), which modify tissue architecture and facilitate transmembrane migration of neutrophils (136). The role of MMPs in TB pathogenesis has been known for some time (137). Several exciting new host-directed therapies against these lung-damaging mediators are being pursued including anti-tuberculosis drugs that also reduce lung damage including the combined use of an eicosanoid inhibitor (used against asthma; zileuton) along with Prostaglandin E2, in addition to other host-directed therapies such as statins, as discussed in the review by Muefong and Sutherland (136).

*Vaccine induced.* Previous publications have highlighted the importance of the innate immune system during BCG vaccination and have indicated that non-specific effects of BCG vaccination may benefit young children even if protection against Mtb is not attained (138–141). A recent study employing depletion and knockout (KO) mouse studies identified that following subcutaneous BCG inoculation, neutrophils, circulating monocytes, and AMs are sufficient to reduce Mtb burden. This study also provided evidence that neutrophils play a significant role in establishing innate immunity, possibly through an early inflammatory response that initiates the reduction in Mtb burden (142). Specifically, depletion of neutrophils in these mouse models was associated with diminished protection by BCG, which supports findings in humans where intradermal BCG was associated with a neutrophil transcriptional signature and elevated neutrophil counts in BCG-vaccinated infants (143). While BCG is effective at preventing disseminated disease in infants, it confers highly variable efficacy against pulmonary TB in adults, particularly in the developing world (12). A greater understanding of the reasons for this variability, together with a better understanding of the early, innate, and non-antigen-specific mechanisms of protection would facilitate the design and development of more effective vaccines (15). Next-generation live-attenuated vaccines based on BCG that aimed to enhance efficacy and durability are well reviewed here (144).

Emerging evidence about the cross-talk between neutrophils and T cells suggests that a balanced targeting of these cell types may improve vaccine-induced immunity (145). While the role of IL-17 in protection against Mtb infection may be related to its role in the induction of TH1 cells (146), IL-17 is also known to activate and recruit neutrophils (147–150). Furthermore, IL-17 favors the recall of vaccine-specific memory T cells in response to Mtb challenge through an IL-23-dependent mechanism (151), and neutrophils when activated by IL-23 also produce IL-17 and IL-22 (152). The role of neutrophils in the induction of specific TH1 and TH17 cells in response to vaccination against TB and the subsequent protective outcome is being investigated. For example, vaccine candidate *M. smegmatis*-Ag85C-MPT51-HspX (mc2-CMX) recombinant live vaccine vector has been shown to elicit a humoral and cellular response against Mtb in mice that is superior to BCG (153), largely due to the TH1- and TH17-specific responses promoted by neutrophils (154). Given the tremendous role of neutrophils in eliciting an enhanced immune response in mice vaccinated with mc2-CMX, neutrophils may represent an underappreciated/bystander innate target for TB vaccine candidates, particularly those targeting POI mechanisms.

### Monocytes/Macrophages

*Effector functions.* Lung resident myeloid cells, including specialized AM, have dual and opposing roles in Mtb control: contributing to host resistance, and a niche for infection (**Figure 1**). Derived from hematopoietic precursors, macrophages are central innate immune cells that function in host defense and maintenance of tissue homeostasis. When tissue homeostasis is perturbed, bone marrow-derived monocytes are recruited from the blood to the affected site where they differentiate into macrophages. Both tissue-resident and monocyte-derived macrophages (MDM) are at the leading edge of an innate immune response through phagocytosis and cytokine release, as well as forming a bridge to adaptive immune responses through antigen presentation (155). Human and animal model macrophages express scavenger receptors and immunoglobulin receptors, which promote phagocytosis (156), antibody-dependent cell phagocytosis (ADCP) (157), and antibody-dependent cell cytotoxicity (ADCC) (158). One of their most robust innate tools are germ-line encoded pattern recognition receptors (PRRs) that sense microbial- or danger-associated molecular patterns (MAMPs/DAMPs). AMs and monocyte-derived macrophages (MDM) are activated by Toll-like receptors (TLRs) TLR2, TLR4, and TLR9 during the early stage of infection with Mtb (159). Furthermore, AM express innate immune receptors, C-type lectin receptors (CLRs), Dectin-1, nucleotide-binding oligomerization domain-like receptors (NLRs), inflammasome-IL-1β activator, and DC-specific intracellular adhesion molecule-3–grabbing nonintegrin (DC-SIGN), for non-specific pathogen recognition and induction of effector functions as observed in *ex vivo* human macrophage studies and surveys of human genetic variants that influence TB susceptibility and outcomes (160–163). Following PRR stimulation, signal transduction cascades converge on the activation of master transcription factors, proteases, and effectors of phagocytosis, allowing for rapid non-specific innate immune responses (156). PRR engagement also ensures durable responses through trained immunity, which is established by metabolic and epigenetic reprogramming of transcriptional pathways in myeloid progenitors (164).

For their role in host defense and inflammatory resolution, activated macrophages from various mouse backgrounds have been divided into two major subgroups from *in vitro* phenotypes. First are classically activated macrophages (M1) associated with inflammatory responses, which can be induced by stimulating resting macrophages with lipopolysaccharide (LPS) and IFNγ. Second, alternatively activated macrophages (M2) are associated with tissue remodeling, resolution of inflammation, and anti-inflammatory responses, and are generated *in vitro* using IL-4 and/or IL-13 (165). While an M1/M2 classification is useful for mapping metabolic pathways of differentially activated macrophages, this does not necessarily reflect the heterogeneity of macrophages present *in vivo*. Macrophages *in vivo* responding to specific external stimuli result in unique subsets that fall between the two extremes of M1 and M2 on a continuum of phenotypes observed in humans (166–168). Metabolic intermediates may have some role as effector molecules like nitric oxide (NO), reactive oxygen species (ROS), and tricarboxylic acid (TCA) derivatives have been shown to regulate macrophage phenotypes and functions by modulating signaling pathways, leading to the production of cytokines, anti-microbial peptides, or tissue repair factors (169).

*Role in Mtb infections.* Macrophages and specifically AM serve as both the first line of defense against Mtb as well as a major intracellular niche for long-term survival in the host (**Figures 1**, **2**). This dual role of macrophages *in vivo* appears to be driven by the cellular activation state. Although a nonselective depletion of macrophages after Mtb challenge in mice improved the outcome of disease, a specific depletion of activated macrophages led to impaired resistance, as reflected by enhanced mycobacterial outgrowth (170). Interestingly, alveolar epithelial type II cells (AECII) have a similar dichotomy in Mtb clearance or protection depending on the progression of infection. While the prevailing dogma is that Mtb is phagocytosed by AMs and dendritic cells once entering the alveolar sac, AECs have also been found to uptake Mtb despite not being professional antigen-presenting cells (APCs). Infected AECs may provide some early innate signaling and cross-talk with innate immune cells, including neutrophils and AMs, and those hypotheses are well discussed here (171). While we focus on the role of AMs in the sections below, it is of note that there is an active and well-warranted study of AECs, and their roles during Mtb infection or control may well be intertwined.

Macrophages are stimulated by PRRs, NLRs, and TLRs to endocytose/phagocytose Mtb and sequester the pathogen in intracellular phagosomes (160, 161, 163, 172). A proinflammatory status activates the intrinsic capacity of macrophages to generate reactive oxygen intermediates (ROI), phagosome maturation, and consequent microbicidal activity (173, 174). Once activated, macrophages in the airway and lamina propria produce a range of pro-inflammatory cytokines and chemokines in response to and in defense against Mtb (160, 161, 175). Within this defense, there is a constant battle of metals going on inside the phagosome of the macrophage between the host cell and Mtb (176). The macrophage delivers an overload of copper and zinc, which are toxic to Mtb deploys a series of protective mechanisms to capture metals, including oxidation and by promoting an increase in metal efflux (176). The upregulation of ctpC gene encoding for the P-type ATPase that regulates the intra-bacterial levels of zinc is another example of how Mtb manages to prevent heavy metal poisoning, observed in an *ex vivo* human macrophage model of challenge (177). As a countermeasure, the macrophage then attempts to block the arrival of nutrients to Mtb, including iron and manganese (176).

A central role of macrophages is the production of TNF and its importance to immunity against Mtb. One of the key pieces of evidence that TNF plays a role in this function comes from the neutralization of TNF in cynomolgus macaques, where TNF neutralization leads to disseminated disease within 8 weeks of infection following adalimumab, also known as Humera^®^ (178). Furthermore, TNF neutralization during latency using a soluble TNF inhibitor, p55-TNFR1, in latently infected nonhuman primates (NHPs) leads to increased lung pathology, bacterial burden, and reactivation (178). Whereas in mice the neutralization of TNF appears to affect the organization of the granuloma leading to lack of control of Mtb (179), the mechanism appears different in NHP, where the granuloma structure does not appear to be impacted (178); however, more neutrophils appear to be present in the granulomas of these NHPs. Immune suppression *via* TNF inhibitors seems to universally increase susceptibility to TB disease across humans and preclinical models. Indeed, in humans, it is well known that the use of TNFa inhibitors are associated with an increased risk of TB disease; therefore, careful screening is applied to individuals requiring treatment for immune-mediated inflammatory diseases needing this treatment course (180).

While IFNγ has been observed to increase survival of mouse bone marrow-derived macrophages by inhibiting bacterial replication (181), it has also been shown to struggle against the ESX system, a sophisticated Mtb secretion system also involved in reducing phagosome maturation (182). Interestingly, host Vitamin D allows the macrophage to increase phagosome maturation (183). In human macrophages, activated Vitamin D3 induces autophagy, as well as direct Mtb killing *via* cathelicidin activation, an antimicrobial peptide that activates the transcription of autophagy-related genes (184). Indeed, previous clinical studies have indicated that Vitamin D deficiency or receptor polymorphisms are associated with an increased risk of Mtb (185–188), and a meta-analysis in 2008 found that low serum Vitamin D levels are associated with a higher risk of active TB (189). A more recent study showed that a serum Vitamin D concentration ≤ 25 nmol/L was significantly associated with an increased risk of active TB, while the range 51–75 nmol/L was not associated with an increased risk of TB (190), suggesting that a threshold of Vitamin D can help promote host anti-Mtb activity.

A strong innate host defense tool against Mtb is bystander macrophages that use efferocytosis for infected cells, which is an efficient means of killing the intracellular bacilli and disposal of debris (191, 192). Apoptosis of Mtb-containing macrophages may result from endoplasmic reticulum (ER) stress and subsequent accumulation of misfolded proteins. ER stress may also induce apoptosis *via* macrophage signaling factors such as inositol-requiring-1α (IRE-1α), double-stranded RNA-dependent protein kinase (PKR)-like ER kinase (PERK), and activating transcription factor-6 (ATF-6) (192). A clinical trial with 185 TB patients showed that clinical Mtb isolates induce autophagosome formation in *ex vivo* macrophages to a variable degree, as measured by LC3II marker protein. Severity of active TB disease in this cohort was inversely related to levels of LC3II production. Collectively, these data indicate a significant protection–mitigation from TB in humans, owing to a macrophage autophagy response (193).

Alternative receptor engagement and immune evasion can lead to different outcomes post macrophage challenge with Mtb. For example, mannose receptor (MR) recognizes mannose present on Mtb, which does not induce bactericidal ROS or phagosome maturation but instead produces anti-inflammatory cytokine signals that help set up a more permissive cell state for Mtb to survive in this intracellular immune niche (172, 174). Mtb can also subvert bactericidal activity in macrophages by escaping the phagosome and persisting in the cytosol of the cell, and specific Mtb antigens can limit or induce macrophage apoptosis and autophagy (174, 182, 194–196). Furthermore, the relatively low dose of challenge with Mtb in normal transmission settings means AM are the first cells infected by Mtb for several days and, in the absence of other inflammatory signals, can be preferentially skewed to promote survival and dissemination, as observed in a mouse aerosol challenge model (197). The dissemination of infected AM from the airway into lung tissues is driven largely by the ESX-1 secretion system in Mtb (197). Work done by Rothchild and colleagues helped demonstrate that AM have an inherent delay in proinflammatory gene transcription post *in vivo* challenge in mice and they instead induce an antioxidant transcriptional program more conducive to Mtb survival (198). Similarly tissue resident macrophages M1 and M2 differentially allow mycobacterial growth, where M2 cells allow more robust Mtb survival than M1 cells, and this seems linked to metabolic profiles in the host (199). Interestingly, Mtb uses cholesterol and fatty acids from the host; therefore, host cells in divergent metabolic states are differentially able to control or support Mtb fitness (199). So, while events following the initial infection of specialized macrophages with Mtb are worth noting, in the absence of activation, metabolic reprogramming, or other proinflammatory signals, these cells become the main permissive hosts for Mtb.

*Vaccine induced.* BCG vaccination has been shown to result in an increase in inflammatory mediators produced by monocytes from healthy volunteers, correlating with histone modification and specific gene activation (138). Indeed, healthy volunteers vaccinated with BCG were observed to have hyper Mtb responsive circulating monocytes 2 and 3 weeks post immunization. This immune potentiation was monitored by secretion of pro-inflammatory cytokines, upregulation of PRRs, and distinct myeloid markers such as CD14, CD11b, and TLR4. Immunized participant PBMCs, when stimulated *ex vivo* with sonicated Mtb lysate, induced a sevenfold increase in IFNγ secretion when compared with basal levels from donors before their BCG vaccination. Furthermore, monocyte secretion of TNF-α and IL-1β was augmented 2-fold. Such trained immune effects associated with histone epigenetic reprogramming depended on the activation of NOD2 receptor, increasing methylation of histone 2 at lysine 4 (H3K4m3) through methyltransferase (138). A follow-up study determined that BCG-trained immunity can be long-lived and effective over 1 year in healthy volunteers’ post-vaccination. Trained monocytes upregulated their PRRs and other innate activation markers, such as myeloid CD14+ TH cells, complement receptor 3 integrin-CD11b+, TLR4, and C-type-1 lectin mannose receptor. These data indicate that non-specific TH cells as well as BCG primed monocytes may collaborate in host protection against Mtb, which help inform and influence vaccine candidate design (200).

To enhance the efficacy, recombinant BCG (rBCG) vaccines (201, 202) are being thoroughly explored and developed. VPM1002, for example, is a rBCG expressing listeriolysin (LLO, encoded by the hly gene used by *Listeria monocytogenes* as a virulence factor for cell to cell spread), which is a clinical stage candidate (28, 203) (**Table 1**). LLO combined with a urease c gene (urec) allows for escape of BCG antigens into the cytosol through perforation of the phagosomal membrane in an optimal pH of 5.5 (urease), promoting MHCI binding of antigens as well as apoptosis induction (203). Another rBCG evaluated in a phase 1 clinical trial was rBCG30 (204). In this case, the strategy was to overexpress Ag85b to increase host immunity to an immunodominant TB antigen. In the Phase 1 clinical trial, both Ag85b-specific CD4+ and CD8+ Th1 immunity in addition to memory T cells were induced and the vaccine was determined to be safe in humans (204). Since this clinical trial, however, rBCG30 has not progressed further.

While BCG is incredibly safe, safer vaccines are needed for those with underlying diseases such as human immunodeficiency virus (HIV), and any acquired or inherited immunodeficiencies where attenuated vaccines can cause unchecked disseminated infection (205, 206). Subunit vaccines, for example, are safe, induce strong antigen-specific immune responses, and have shown promise against TB disease in humans. Protein antigens, which are, on average, weak immune stimulants, are partnered with immune driving adjuvants that classically target PRR signaling pathways that are abundant on macrophages (207, 208). Several subunit vaccines are currently in the clinical pipeline (19, 209), and the recent success of candidate M72+AS01_E_ has provided confidence that an adjuvanted subunit vaccine is a feasible approach for prevention of TB disease (13, 14). The AS01_E_ adjuvant includes bacterially derived monophosphoryl lipid A (MPL) from *Salmonella minnesota*, in addition to saponin QS-21 (210). Vaccinating mice with AS01 directs monocytes to help dendritic cells as well as activation of adaptive immune players like CD8+ T cells or NK cells (57). Another promising adjuvant contains a synthetic version of MPL, glucopyranosyl lipid adjuvant (GLA), that, when combined with a stable emulsion (SE) and ID93 antigen, constitutes a candidate with documented efficacy in several animal models including mice, guinea pigs, and NHPs, and is in late Phase 2 clinical trials (40–49, 211, 212). The mechanism of action of GLA-SE has been previously described, where its drive of innate immune responses *via* TLR4 promotes the generation of antigen-specific TH1 CD4+ T cells in mice (50, 51, 213, 214). Another candidate subunit vaccine in the TB vaccine clinical pipeline is H56:IC31 (215). IC31 is a two-component adjuvant that contains a KLK antimicrobial synthetic cationic peptide vehicle along with TLR9 agonist phosphodiester-backboned oligodeoxynucleotide (ODN1a), which induces type 1 IFN (69). Unlike the MPL-based adjuvants discussed above, in mouse models of vaccination, IC31 has been shown to induce both TH1 and TH17 polarizing T-cell response (216–218). While these subunit vaccine adjuvants (AS01_E_, GLA-SE, and IC31) drive some innate responses in macrophages, their main influence is on dendritic cells (51, 69, 218), which will be discussed at length below (**Table 1**). In addition to adjuvant strategies designed to drive innate immune responses, Mtb antigen selection in vaccine candidates can also be used to combat Mtb immune evasion tools specifically at the macrophage. For example, ESAT-6 is a well-studied candidate antigen included in subunit vaccines, and is encoded by esxA in genetic locus region of difference 1 (RD1) (219). ESAT-6 has been shown to inhibit autophagic flux by impeding autophagosome–lysosome fusion in *ex vivo* cell culture, which assists in Mtb immune escape from macrophages (220–222). Preclinical studies in Balb/c mice show that vaccination with ESAT-6 and c-di-AMP regulate macrophage autophagy, resulting in the inhibition of Mtb growth in macrophages during early infection (223).

Beyond vaccination regimens, therapeutic-based strategies are being developed to target macrophages and overcome metabolic barriers to Mtb killing in these cells (224, 225). There is growing interest in the induction of autophagy in Mtb-infected host cells using autophagy-inducing compounds (AICs). Nanoparticles (NPs) can be utilized as a delivery system to improve the activity of AICs against intracellular Mtb by transporting the encapsulated AICs to their target sites while protecting them from biodegradation and enhancing their absorption across biological barriers (226, 227), which has been demonstrated nicely for delivery of rifampicin to macrophages *in vitro* (227). NPs sustain drug release in the target tissues, allowing for the reduction of dosing frequency and lessening drug-associated side effects in the process (228). In addition, the materials used to synthesize the NPs can possess autophagy-inducing activity or the surface of the NPs can be integrated with an AIC (229). Upadhyay et al. reported the capacity of yeast-derived glucan particles (GP) loaded with high payload of rifabutin (RB) NPs [(RB-NPs)-GP] to induce anti-mycobacterial and cellular activation responses in Mtb-infected J774 macrophage cells. The exposure to (RB-NPs)-GP triggered strong innate immune responses in Mtb-infected macrophages including the induction of apoptosis, autophagy, as well as ROS and NOS. Macrophage targeting particles containing rapamycin are also in development to induce Mtb killing in infected cells *via* mTOR inhibition to drive autophagy, and have shown promise as spray dried formulations delivered to mice (225, 230). Careful examination of bacterial evasion and host regulation can lead to more promising candidates for targeted therapies. For example, the absence of Alox5 leads to increased CD8+ and CD4+ T-cell responses in mice by cross-presentation and MHCII antigen presentation pathways (231), which may be carefully exploited. However, additional investigations *in vivo* as well as studies to target and deliver known AICs are required to advance these therapies (232).

### Dendritic Cells

*Effector functions.* Dendritic cells (DCs) are considered APCs and immature or less differentiated DCs are present in many tissues. Like macrophages, DCs use PRRs to survey for potential antigenic material and leverage phagocytosis as well as macropinocytosis, receptor-mediated endocytosis, or direct contact with damaged or infected cells (233–235). In contrast to other phagocytes, once DCs receive activation signals resulting from pathogenic interaction or inflammatory stimuli, they mature and migrate to secondary lymphoid organs for antigen presentation (236) (**Figure 2**). Antigens can be prepared by lysosomal proteases within the endocytic pathway or proteosomes for MCH I or II presentation, allowing for both naïve CD4+ and CD8+ T-cell priming (236). Additionally, endocytosed proteins can be cross-presented *via* MHCI (235, 237). Activated DCs serve as a bridge between innate immune surveillance and adaptive immune activation.

In both humans and preclinical mouse models, DCs are observed to be a heterogeneous population containing subsets with unique surface markers and specialized functions (238). There are two overarching subsets to which human and mouse DCs are traditionally characterized: conventional DCs (cDCs) and non-conventional DCs (239). While certain markers and subsets of human and mouse DCs may differ, their general functions are similar. Conventional DCs, derived from conventional DC progenitors, are characterized by their APC functionality and can be found within lymphoid tissues and barrier tissues (lung, skin, intestinal tract, kidney, and liver) (240, 241). Further development of cDCs is directed by transcription factors differentiating the cDC1 subset, which cross present to naïve CD8+ T cells, or cDC2 subset, with focused presentation to naïve CD4+ T cells (240–245). Plasmacytoid cells (pDCs), derived from common DC progenitors, largely encompass the non-conventional DC subset. pDCs primarily circulate in the blood and secondary lymphoid organs, but also localize to skin, intestine, and epithelial tissue during inflammation (246). pDCs are characterized by large production of type I IFNs during viral infection, and when activated, pDCs secrete TNF-α, IL-1, and IL-6 (246).

*Role in Mtb infections.* DCs are critical for initiating anti-Mtb responses as primary APCs that present antigens and induce adaptive immunity. From the bone marrow, DC progenitors enter the bloodstream and home to different epithelial tissues, including airway epithelium and lung parenchyma, the primary site of Mtb challenge. Like macrophages, human DCs are observed to use a series of PRRs to bind and phagocytose Mtb in *ex vivo* co-culture conditions; this includes complement receptors, MR, TLRs, and DC-SIGN (247–249). Mtb uptake by DCs leads to DC maturation and upregulation of MHCI, MHCII, CD40, CD54, CD58, and CD80 (248). After reaching draining-secondary lymphoid organs, DCs present antigens to prime naïve T cells activating Mtb-specific responses and effector differentiation (**Figure 2**). While this is the common response to infection, studies have shown that DC activity may vary depending on Mtb strain. Transcriptional analysis of human monocyte-derived DCs (moDCs) infected with different Mtb strains found that infection with Mtb H37Rv led to significant upregulation of EBI3 and, compared to other mycobacteria, induced the least level of IL-10 expression (250). In this same *ex vivo* moDC challenge model, BCG-Japan induced CCR7 and TNF-α expression and showed considerably lower intracellular growth, suggesting that DC responses reflect challenge strain virulence. Corroborating these findings, a study by Ramos-Martinez et al. reported that human moDCs infected with a hypervirulent lineage 3 Mtb strain neutralized intracellular growth of the bacilli, underwent decreased apoptosis, and led to poor T-cell expansion compared to an Mtb H37Rv reference strain (250). In contrast, human moDCs infected with hypovirulent lineage 4 strain displayed high apoptosis and consequently this precluded their capacity to expand T cells. The relationship between host cell responses and immune evasion strategies by virulent mycobacteria is still an active area of research. However, as DCs help drive adaptive immunity, they are a logical target for specific vaccine strategies, which are discussed below.

*Vaccine induced*. Robust T-cell responses following vaccination is vital for successful durable immunity against Mtb. Delayed or low T-cell activity in the mouse model has been observed and suggested as a bottleneck for protection following vaccination (251). In order to overcome this delay, researchers evaluated the adoptive transfer of stimulated DCs intratracheally into BCG vaccinated mice and observed that this group had a ~1.5 CFU log reduction compared to BCG vaccinated only controls (252). Transfer of activated DCs into non-vaccinated mice saw a reduction in lung CFU comparable to the vaccinated controls, demonstrating the profound influence of DCs in the correct location on maturing a protective immune response. While mechanistically informative, adoptive transfer of activated DCs is not a robust strategy for global TB protection, but certain vaccine approaches instead preferentially target DC activation. For example, one strategy is the selection of DC-activating proteins into a recombinant vaccine (253). The mycobacterial heat-shock protein Rv2299c (heat-shock protein 90 family) in mice leads to DC maturation *via* the TLR4 pathway, with TH1 leaning towards cytokine production and enhanced expression of MHCI and MHCII. Administration of Rv2299c fused with the T-cell-stimulating antigen ESAT-6 following BCG immunization in mice showed significant reduction in lung bacterial burden and inflammation compared to BCG control 16 weeks post challenge with Mtb HN878. This study helps to underscore the importance and promise of DC activity particularly in multiantigen vaccine designs. Additional studies have investigated other novel antigens that target DC maturation and enhance TH1 responses, including a DosR regulon-encoded protein (Rv2005c) fused with macrophage-activating protein, Rv2882c, and Rv1876 (254, 255). Common to these mouse immunological studies is enhanced DC maturation, increased expression of IFNγ, IL-2, and nitric oxide, and decreased bacterial burden. Given the importance of DCs for priming T cells and in turn robust T-cell activity for protection, new vaccine approaches have wisely targeted DCs *via* antigen selection and other vaccine components to boost downstream responses.

DCs are also targeted through use of immunogenic adjuvants. For example, clinical stage saponin-based adjuvant, AS01, composed of QS21 and TLR4 agonist MPL, leads to release of alarmins and subsequent DC activation in mice (57). This study of acute responses in mice after immunizations found that the addition of AS01 to antigen led to an 8.6-fold increase of MHCII+ DCs within the draining lymph node compared to antigen alone, as well as enhanced T-cell priming, making DCs a crucial target of the AS01-induced immune response (57). AS01_E_ is being utilized in human vaccines including the shingles recombinant zoster vaccine (Shingrix), the RTS,S malaria vaccine, as well as the M72 Mtb vaccine candidate. In a study that analyzed the RNA expression in PBMCs and whole blood from participants who had received the M72/AS01_E_ vaccine, researchers found that these samples were enriched in activated dendritic cells compared to baseline measurements up to 31 days post-injection (256). Despite the multiple safety and immunogenicity studies completed for the M72/ASO1_E_ candidate, there are little data phenotyping the vaccine-induced DC response in humans. Following the M72/AS01_E_ vaccine candidate was the release of a similar protein-adjuvant strategy leveraging vaccine candidate ID93+GLA-SE. Early studies on GLA showed its ability to stimulate both mouse and human DCs leading to high production of TNF, IL-6, and IL-12p40 (51). Administration of GLA-SE through intradermal injection of human skin in an explant model showed that DC enhanced CD4+ T-cell proliferation (257). Indeed, three major subunit vaccine adjuvants (AS01_E_, GLA-SE, and IC31) rely on DC activation for optimal protective responses in candidate vaccines as they are the most efficient APCs for adaptive immune responses (51, 69, 218).

As for DC responses to BCG, which is currently the only licensed TB vaccine, an *in vitro* study found that BCG-infected human DCs produced TNF-α, IL-1β, IL-6, and IL-10 (258). However, when BCG-DCs were co-cultured with human cord-blood mononuclear cells, they produced anti-inflammatory cytokine IL-4, which may partially contribute to low protection garnered by BCG. In the elderly, a study surveying whole blood and plasma samples 1 month post BCG immunization observed enhanced plasmacytoid and myeloid DCs (259). Despite this, plasma levels of type I IFNs (IFNα/β) were significantly decreased, while type III IFNs (l), which comprise anti-viral cytokines (also named IL-28A, IL28B, and IL-29), were significantly enhanced, potentially showcasing the off-target effects of BCG (259). Next-generation BCG vaccine candidates may also work to improve and direct BCG-induced inflammatory DC activation as a proposal for enhanced protection.

### Natural Killer/NKT Cells

*Effector functions.* Natural killer (NK) cells are lymphocytes within the innate immune system harboring potent cytotoxic effector functions. NK cells help control diseased states by identifying and attacking infected or malignant cells directly through cellular toxicity and indirectly *via* cytokine signaling (260). NK cell activation is regulated by activating inhibitory cell surface receptors (261, 262). MHCI expressed by healthy nucleated cells bind NK inhibitory receptors [killer cell Ig-like receptors (KIRs), leukocyte Ig-like receptor (LIR), and CD94/NKG2 receptors] and prevent cytotoxic activity (262). Conversely, infected cells downregulate surface MHCI and upregulate co-stimulatory ligands to engage NK cell-activating receptors. The cytokine milieu induced downstream by other activated innate cell subsets can also direct and help influence NK cell activity. *In vitro* studies with mouse and human-derived cells have shown enhanced NK cell activation from DC-produced IL-12, IL-18, IL-15, and IFN-α/β (263, 264).

Once activated, NK cells have multiple mechanisms of cytotoxicity that are largely shared with CD8+ T cells, including release of granzymes, perforin, and utilization of death receptors (265, 266). Perforin, a glycoprotein, binds the cell membrane of the target cell and forms pores (267). The pores can disrupt the cell membrane and subsequently destabilize mineral homeostasis, which can indirectly induce apoptosis. Additionally, perforin can aid in other targeted cytotoxic effects, including the entry of granzyme through perforin-formed pores (268, 269). Granzymes, a family of serine proteases, cleave proteins such as procaspases, which initiate intrinsic apoptotic signaling cascades and induce target cell death (270). NK cells can also induce extrinsic apoptotic signaling *via* two primary TNF receptors, Fas (CD95) and TNF-related apoptosis-inducing ligand (TRAIL) (271). Binding of Fas, widely expressed on tissues, and Fas ligand, expressed by activated NK cells and cytotoxic T lymphocytes, results in nuclear condensation, membrane blebbing, caspase activation, and eventual cell death (272). Similarly, cross-linking of TRAIL, and TRAIL-R1 and -R2, leads to caspase activation and apoptosis (273–275).

In addition to cytotoxic effects on infected cell targets, NK cells can regulate immune responses *via* T-cell activity, indirectly *via* IFNγ signaling or directly *via* cytotoxic-mediated killing (276). *In vitro* and *in vivo* studies have found that IFNγ produced by NK cells promote DC production of IL-12, leading to downstream enhanced CD8+ T-cell activity (277, 278). In addition, NK cells can regulate T-cell activity indirectly *via* IFNγ signaling or directly *via* cytotoxic-mediated killing (276). *In vitro* studies of human cells and *in vivo* mouse models have found that IFNγ produced by NK cells promote DC production of IL-12, leading to downstream enhanced CD8+ T-cell activity (277, 278). IFNγ signaling also is known to promote CD4+T cell TH1 differentiation (276, 279–281). Beyond downstream cytokine activity, both *in vivo* and *in vitro* studies have shown that NK cells can target T cells *via* secretion of cytolytic granules or death receptors (282–284).

*Role in Mtb infection*. As discussed above, NK cells have multiple roles in driving immune responses, illustrating their importance in combatting infection. While NK cells have been known to play a large part in immune responses to viral infections, there has been growing interest in the role NK cells play during Mtb infection (**Figure 1**). *In vitro* studies have found that upregulation of stress-induced UL-16 binding protein by Mtb-infected human monocytes leads to recognition by NKG2D-activating receptor followed by NK cell-mediated lysis (285, 286). Interestingly, while granzyme A-producing NK cells are observed after challenge with Mtb in an *in vivo* mouse challenge model, it did not correlate with protection from bacterial burden (287). These data together may highlight the differences between models, or suggest many compensating pathways derived from NK cells that contribute to Mtb control. For example, NK cells have been observed to restrict intracellular Mtb growth in infected human monocytes through direct contact and partially mediated through secretion of soluble factors such as IL-22 and IFNγ (288–290). *In vivo* studies in C57BL/6 mice infected with Mtb showed that NK cell depletion had no significant effect on bacterial burden (291). However, T-cell-deficient Rag mice with further NK cell depletion demonstrated enhanced bacterial burden post challenge, which may highlight the importance of NK cells in immunocompromised environments and possible overlap of NK and cytotoxic T-cell functions that make parsing out direct contributions difficult. NK cells are also found in later stages of Mtb infection and have been identified to infiltrate granuloma lesions in Mtb-infected patients (292). The direct influence of NK cells, like neutrophils, may depend on their location and timing with respect to challenge with Mtb.

Adjacent to NK cells are NK T cells, which co-express the T-cell receptor (TCR) and NK cell surface receptors (293, 294). This co-expression confers both adaptive and innate immune activity (294). Invariant NKT cells (iNKT) are the primary subset of NK T cells identified. iNKT cells recognize self and foreign lipids presented by the glycoprotein CD1d (295). As summarized by Ruibal et al., there has been growing interest in iNKT cells’ role in controlling Mtb infection (296), largely due to the observation that iNKT cells have increased activated phenotypes in TB patients compared to LTBI and healthy controls (297). Additionally, the number of NKT cells measured in patients during TB diagnosis correlated with faster responses to antibiotic treatment (298). Given these dichotomies of NK/NKT cell activation and numbers in different patient populations and outcomes, they may serve as an important predictive correlate for TB protection and should be examined closely in vaccine candidate evaluations.

*Vaccine induced*. NK cell’s ability to target infected cells and regulate T-cell activity has enhanced interest in vaccine-induced NK cell responses. An *in vivo* study found that C57BL/6 mice immunized with BCG and subsequently challenged with Mtb H37Rv showed increased IFNγ- and IL-22-producing NK cells compared to controls (299). To further determine if these cells played a role in protection afforded by BCG, researchers treated mice with 0.3 mg of anti-NK1.1 for NK cell depletion during BCG vaccination. This NK depletion led to an increase of immunosuppressive T-regulatory cells, increase in bacterial burden, and reduced T-cell activity after Mtb H37Rv challenge. However, supplementing with IL-22 restored some BCG-induced protection, exhibiting the importance of the IL-22–NK cell axis in protection (299). Unfortunately, NK cells have been an underappreciated subset of cells that should be evaluated in POI and POD vaccine candidate screens.

### B Cells

*Effector functions.* B cells are an immune target of many vaccines, and their main effectors, antibodies, are crucial particularly for viral and extracellular bacterial infections. B cells are, however, also important for protection against intracellular bacteria including Mtb as reviewed in depth by Chan et al. (300). T-cell regulation, for example, can be geared towards regulation or different helper T-cell phenotypes through cytokines produced by B cells. Like T cells, B cells are also classified into different subsets: B effector 1 (Be1) producing IFNγ, IL-12, TNF, IL-10, and IL6; B effector 2 (Be2) producing IL-2, lymphotoxin, IL-4, IL-13, IL-10, and IL-6, as reviewed previously (300).

The effector functions of B cells during tuberculosis infection are several-fold as discussed in a recent review by Rijnink et al. (301). These properties include the production of antibodies, antigen presentation, as well as cytokine production. Antibodies, or immunoglobulins (Ig), can be cell bound or secreted, make up five classes or isotypes (IgG, IgA, IgE, IgM, and IgD), and have numerous functional capabilities due to the interaction with other immune cells *via* constant fragment receptors (FcR) and components that facilitate the host response to infection. Since these B-cell and antibody functions have been extensively reviewed in the context of tuberculosis by others in the field (301–303), this section is only meant to serve as a broad overview and highlight specific findings.

B cells, which constitutively express major histocompatibility complex class II (MHCII), are in the “professional” APC category along with DCs, monocytes, and macrophages (304). As eloquently described in the review by Ghosh et al., once a foreign antigen is recognized by the B-cell receptor (BCR), this leads to internalization and ubiquitination steps that transport the Ag-BCR clusters to MHCII loading compartments within the cell in either endosomes or lysosomes. The MHCII molecule assembles in the endoplasmic reticulum along with trimers of invariant chain (Ii; CD74), which, when Ii is processed a place-holder peptide (class II invariant chain peptide, or CLIP), binds into the MHCII groove. Following a number of processes outside the scope of this review, the antigen undergoes proteolytic processing followed by a loading of higher-affinity peptide from the pathogen (or other immunogen) into peptide/MHCII complexes, displacing CLIP. These complexes are exported to the surface of the cells and presented to CD4+ T cells (304). B cells present antigen to T follicular helper cells (Tfh), and subsequent differentiation of Tfh is dependent on B-cell cytokine production following stimulation from pathogens. Antigen presentation on B cells can also impact T-cell memory responses. Our group has shown that B-cell-deficient mice fail to generate vaccine (ID93+GLA-SE)-induced MPEC (memory “precursor” effector CD4+ T cells) in mice, and this deficiency reduces memory T-cell recall responses (305). This is in alignment with other studies that have shown evidence for decreased T-cell responses or protective immunity against intracellular pathogens in animal models with B-cell deficiencies (306–310).

*Role in Mtb infections.* There are several animal models, particularly mouse models, and experimental studies that have helped elucidate the effects of immune cells on the outcome of Mtb infection. The use of B-cell-deficient mice for understanding the role of B cells and antibodies has been reported by several investigators, utilizing several different Mtb strains, doses, and routes of infection, all leading to different conclusions regarding the importance of B cells and/or antibody responses on early, chronic, or disseminated TB [reviewed here (301)]. As increased age has been associated with recrudescence of TB, Turner et al. investigated the progression of TB disease in the aerosol model using Mtb Erdman challenge in B-cell-disrupted C57BL/6j-IgH-6 mice, IL-4 gene-disrupted mice, or in wild-type mice (311). An aging immune response results in increasing numbers of B cells and Th2-biased responses. In this study, bacterial burden was similar in the lungs of the mice, regardless of the defect. In an acute Mtb mouse study done in B-cell-deficient mice, infection with Mtb CDC1551 resulted in a delay in bacterial dissemination to the spleen and liver, and decreased lung pathology (312). In contrast, in another study performed in B-cell-deficient mMT mice infected with Mtb Erdman, researchers observed increased recruitment of neutrophils and worsened immunopathology, and increased IL-10 within the lung, suggesting that B cells contribute to the modulation of inflammation and enhancement of immunity against infection (313). With an increased infectious dose of Mtb Erdman (300 CFU), B-cell-deficient mice were slightly more susceptible to infection (40% mortality compared to 0% mortality in the wild-type mice), demonstrated increased pathology within the lung, had increased neutrophil numbers in the lung, and enhanced IL-10 levels as well in the lungs. Vordermeier et al. also demonstrated that, in µMT B-cell-deficient mice given high-dose (10^6^/mouse in 0.1 ml) i.v. injection of Mtb H37Rv, B cells play a role in containing infection in the lung, spleen, and liver (313), although higher mortality was not shown in the B-cell-deficient mice over 18 weeks following infection (data not shown, but communicated in the manuscript). In addition, vaccination of the B-cell-deficient mice with BCG 4 weeks prior to challenge with Mtb provided protection, suggesting that T-cell responses were not impaired over that short period of time.

*Vaccine induced.* The design of preclinical vaccines against µtb has typically relied on ways to generate a potent TH1 cellular response. The correlates of vaccine efficacy against tuberculosis are unclear, however, and attempts to determine these correlates could aid in developing a vaccine that provides long-lived protection on its own or as a vaccine that suitably boosts BCG. While few dispute the benefits of generating a cellular response as a requirement for immunity against TB, B cells and antibodies have convincingly shown beneficial protective effects against TB. Recent research has shed additional light on the role of B cells against TB and may in fact warrant further investigation particularly during vaccine development (**Figures 1**, **2**). A lot of attention has recently focused on the results of superior (nearly sterilizing) protection following i.v. BCG vaccination in rhesus macaques (16), where the model has suggested prioritizing interesting correlates of protection. The antibody responses induced from i.v. BCG immunized rhesus macaques were thoroughly investigated (17). In this study, significantly enhanced IgG1, IgA, and IgM titers were observed against lipoarabinomannan (LAM) in the lungs (bronchoalveolar lavage) 4 and 16 weeks after i.v. immunization, and increased anti-LAM IgG1 and IgA responses at 8 and 24 weeks, and higher anti-LAM responses 8 weeks following i.v. immunization in the plasma, all compared to i.d. administration. In addition, various antibody responses to HspX and the glycoproteins PstS1 and Apa were also elevated in the i.v. BCG-immunized NHPs compared to the standard i.d. route. Enhanced functional antibody responses in the BAL 4 weeks after immunization was also importantly demonstrated, including an increase in FcγR2A binding, NK cell degranulation, and intracellular antibody-dependent Mtb killing within macrophages with i.v. immunization in these studies (17). Lastly, plasma LAM-specific IgG1 (week 8) and LAM-specific IgM (week 24), in addition to HspX-specific IgM (week 4), were predictive of vaccine-induced protection (<1,000 CFU) using partial least-squares discriminant analysis. A comprehensive analysis of the role of anti-LAM antibodies is well reviewed here (314). While i.v. BCG is not being considered as a viable route of immunization, these findings are valuable indicators of potential humoral correlates of protection that can be tested for other preclinical candidates and clinically in historical or planned trials.

The route of immunization can also play a role in vaccine-specific antibody responses. Heat-killed MTBVAC (MTBVAC HK) delivered mucosally in mice (*via* the intranasal route) and in NHP (intrabronchial route) induces mucosal IgA, IgM, and IgG responses (315). When BCG-primed C57BL/6 mice were given intranasal immunization with MTBVAC HK, enhanced survival following high-dose Mtb H37Rv was observed compared to BCG (given s.c.) alone. One month following MTBVAC HK immunization, antigen-specific IL-17 in addition to IFNγ was induced in the lung and spleen. Our group has also shown that mucosal delivery with ID93+GLA-SE leads to antigen-specific IL-17 production and increased protection 4 weeks after the last immunization; however, both intramuscular and intranasal delivery led to increased protection in the lungs of immunized mice infected with low-dose Mtb H37Rv (52). Although antibody responses were not measured following mucosal delivery, i.m. immunization with ID93+GLA-SE routinely induces antigen-specific IgG2 and IgG1 responses, and induces TH1 CD4+ T cell cytokine responses (IFNγ, TNF, and IL-2) (40, 42).

A correlate of vaccine-induced protection against Mtb would enable down-selection of vaccines that enter into the Mtb vaccine clinical pipeline and would accelerate lengthy clinical trials, and antibody responses should not be overlooked in defining these correlates. A United Kingdom (U.K.) study conducted over 10 years showed that people with hypogammaglobulinemia have an increased risk of contracting Mtb (316). Several different functional roles of antigen-specific antibodies against Mtb have recently been described by the Alter lab in Lu et al (303). Fc-mediated effector responses are dictated by the interactions of the Fc domain of the antibody with the activating or inhibitory Fc receptors on innate cells such as NKs, monocytes, and neutrophils (303). PPD-specific responses from people latently infected with Mtb had higher PPD-specific NK cell enhancement, ADCC, and NK cell degranulation in comparison to those with active TB. Different antibody Fc effector profiles were also shown to correlate with different states of TB disease utilizing a systems serology approach. Antibodies may hold the key for biomarker indicators that correlate with TB disease, providing an efficacy screen for treatments, which, in turn, could help reduce the spread of Mtb.

Different TB-specific antibody subclasses and glycosylation patterns have been characterized, offering interesting perspectives on how a reduction in a humoral marker of TB disease (IgG4) can potentially track the success of drug treatment (317). Grace et al. have recently reported striking differences in the TB-specific antibodies in four human cohorts in different stages of TB: healthy, latent TB infection (LTBI), active TB (ATB), and treated active TB (txATB) (317). ATB subjects were enrolled within 1 week of treatment with isoniazid, rifampicin, ethambutol, and pyrazinamide for 2 months followed by 4 months of isoniazid and rifampicin. The treated cohort were patients that completed 6-month antibiotic treatment, and were culture negative at 2 and 6 months of therapy. LTBI patients were contacts of patients with ATB, with positive QuantiFERON-TB Gold results but no symptoms of TB. Antibody profiling included responses to several TB antigens including PPD, recombinant Ag85A/B (1:1 ratio), ESAT6/CFP10 (1:1 ratio), GroES, glcB, and HspX (317). Of note, enrichment of PPD-specific IgG4 was shown in the ATB cohort, and depleting this specific subclass led to increases in neutrophil and NK effector functions. In addition, this marker of TB disease (PPD-IgG4) was decreased in the txATB cohort. Besides the enrichment of PPD-specific IgG4 in ATB, higher levels of Ag85-specific IgG were observed in the ATB cohort. In the treated group, PPD-specific phagocytosis, and increased HspX-IgG and Ag85-IgM were seen in the txATB subjects. In the ATB group, expanded IgA titers to Ag85A/B whereas higher PPD-IgM and HspX-IgG1 were significantly higher in the txATB cohort, which may be indicative of effector functions used to control infection (317).

One vaccine candidate success story warranting further humoral endpoint evaluations is the M72+AS01_E_ clinical trial, which has shown ~50% efficacy against the development of pulmonary TB disease (13, 14). For humoral immunity, only the geometric mean anti-M72 IgG antibody responses were assessed, where the participants were seropositive at 2 months (highest responses) and had detectable anti-M72 titers through 36 months (13). Given the IgM correlation described above in NHPs, it will be of interest to measure anti-M72 IgM responses in these cohorts.

Another success story is that of ID93+GLA-SE. This vaccine is currently in Phase 2 and has shown promise both for safety and immunogenicity (45). In the phase 1 trial, all ID93+GLA-SE recipients showed significantly greater ID93-specific IgG titers after one injection compared to the protein (ID93) alone, and after three vaccinations, these responses persisted until Day 238. There was a predominance of anti-ID93 IgG1 and IgG3 subtypes. Anti-ID93 IgM responses were also measured following immunization. Interestingly, stronger IgM responses were observed against ID93, with some anti-IgM response against Rv2608 and Rv1813, and low IgM responses to Rv3620 and Rv3619 components of ID93 in the ID93+GLA-SE-immunized cohorts. Antibody effector functions were also measured in ID93+GLA-SE-immunized cohorts, including antibody-dependent cellular cytotoxicity (ADCC), and ID93-specific antibody-mediated NK cell degranulation and activation determined by the enhanced IFNγ, MIP1β, and CD107a upregulation. In addition, antibody-mediated cellular phagocytosis (ADCP) of ID93-coated beads was increased from the sera of those vaccinated with ID93-GLA-SE compared to ID93 alone (sera was analyzed 28 days after the third immunization). Antibody functions in this study were correlated with multiple subclasses and isotypes rather than one. These responses also suggest that the adjuvant, GLA-SE, is able to augment antibody effector functions as these responses were not elicited in the ID93 protein alone group. To our knowledge, this was the first candidate TB vaccine that was analyzed for antibody effector functions. Specific antibody-mediated immunity is well reviewed here (301, 318) and provides ample rationale of why these endpoints should be expanded for clinical TB vaccine candidates.

### CD8+ T Cells

*Effector functions.* T cells expressing CD8 are largely known as killer T cells with effector functions related to T-cell receptor (TCR) engagement by antigen presented on MHC class I molecules, helping to fight intracellular pathogens. CD8+ T cells have an arsenal of cytotoxic molecules, including Fas ligand (binding Fas [CD95] on target cells and inducing apoptosis), perforin (driving membrane holes in target cells), and granzymes (protease enzymes that induce target cell apoptosis and usually enter perforin-induced holes) (319). Perforin and granzyme are stored in lytic granules at the ready, while other membrane-associated receptors and cytokines are produced *de novo* upon receptor binding. Activated CD8+ T cells express proinflammatory cytokines, notably IFNγ and TNF family members (319), as well as paracrine and autocrine proliferation inducing IL-2, which combine to help activate local and distal responses.

After activation, human CD8+ T-cell expansion and contraction are regulated by distinct changes in metabolic pathways and cell death induction (320), namely, restimulation-induced cell death (RICD) (321) and cytokine withdrawal-induced cell death (CWID) (322). Those cells that survive these expansion and contraction phases make up the memory compartment, which is leveraged for faster subsequent pathogen encounters, the hallmark of adaptive immunity. Memory CD8+ T cells comprise a spectrum of subset lineages with variable longevity, localization, and reactivity, which are well reviewed here (323). Given their role in combating intracellular pathogens and ability to form T-cell memory, CD8+ T cells and their effector functions have been well studied in Mtb infection and vaccine-induced immunity (321).

*Role in Mtb infections.* Mtb antigen-specific CD8+ T cells have been isolated post challenge in preclinical models and can migrate to the lung post infection in both preclinical and clinical evaluations (324–326), induce IFNγ and lyse Mtb-infected macrophages in vitro (327–331). Depletion or disruption of either MHC class I or CD8+ T cells significantly enhances Mtb susceptibility in mouse models, and IFNγ derived from CD8+ T cells contributes to controlling bacteria in vivo (331–335). In mice, granzyme A (GZMA)-producing CD8+ T cells are observed after challenge with Mtb in vivo (287); however, GZMA deficient (GZMA-/-) mice are not more susceptible to Mtb infection or TB morbidity than wild-type mice. These data suggest that while GZMA may play a role in infection and immunity, there may be other pathways that compensate in its absence, and indeed candidate vaccine MTBVAC-induced protection was not reduced in GZMA-/- mice compared to wild-type mice (287). Importantly, CD8+ T cells generate pulmonary immune memory and can be activated post challenge, as demonstrated by a C57BL/6 mouse model of i.v. Mtb infection, drug treatment, and Mtb aerosol rechallenge (329). It may be that the contribution of CD8+ T cells in controlling Mtb has been underestimated because they are more involved in the latent phases than acute phases of infection and depletion studies may have lacked the dynamic resolution to study these kinetics (**Figures 1**, **2**). In an exceptional murine study design from Pinxteren and colleagues, depletion of CD8+ T cells during the acute stages (days 1–21 post challenge) of infection did not result in higher bacterial burden, whereas depletion during a latent phase (11 weeks post drug treatment) increased pulmonary bacterial burden 10-fold (336).

In a screen of human cohorts with positive tuberculin skin tests (TST), T cells were stimulated with synthetic peptide pools (337) and 74 different Mtb proteins, 58 novel, were determined by IFNγ ELISPOT to have CD8+ immunodominant antigens (338). In addition to antigen recognition, *ex vivo* human Mtb-antigen specific CD8+ T-cell lines lyse Mtb-infected macrophages and inhibit intracellular persistence (327). Antigen specificity and localization may indeed be critical components of CD8+ T-cell vaccine designs as a cohort of TB-infected individuals from the Gambia showed reduced CD8+ T-cell activation and cytotoxicity by flow cytometry after stimulation with Mtb H37Rv compared to healthy BCG-vaccinated controls (326). While the overall percent lysis of target cells infected with Mtb H37Rv *ex vivo* was equal between cohorts, the TB-infected group did have a significantly higher specific cell lysis against target cells with recombinant vaccinia virus (rVV)-ESAT6 compared to healthy controls (326). Furthermore, the specific phenotype of CD8+ T cells has been observed to track with progressive disease where *ex vivo* stimulated CD8+ T cells from participants with active TB disease have increased TGFβ and IL-10 and decreased granzyme B expression that correlates with bacterial load in induced sputum samples (339).

CD4+ T cells are decidedly not the focus of this review; however it is critical to note their role in maintenance of other cells, including CD8+ T cells across a variety of infectious disease models (340). Interestingly, Lu and colleagues recently demonstrated that in a mouse model, CD4+ T cells provide help to CD8+ T cells, reducing exhaustion and improving control of Mtb *in vivo (* 341). These studies suggest that the relative contribution of CD8+ T cells in CD4+ preclinical KO experiments may have underestimated their role. Furthermore, with the advancement of *in situ* analyses, single-cell evaluations, and TCR sequencing, we will further unravel the complexities of T-cell subsets and their role(s) in protection and disease. For example, recent single-cell analysis of TB pleural effusion in humans demonstrated that CD8+ T cells expressing GRZM K are enriched in pleural fluid and may contribute to this disease state (342). Balance of CD8+ T-cell phenotypes, localization, and abundance are likely important for driving immunity versus pathogenesis.

*Vaccine induced.* While RNA vaccine platforms (343) are in development for TB vaccine candidates, many vaccine strategies designed to induce robust anti-Mtb CD8+ T cells have been evaluated and are well reviewed here (344–346). These platforms include recombinant BCG encoding listeriolysin O (VPM1002), recombinant adenoviruses containing Ag85A (Ad5Ag85A) and Ad35 containing Ag85A, Ag85B, and TB10.4 (Aeras-402), chimpanzee adenoviral vectored vaccine (ChAdOx185A), recombinant vaccinia virus (rVV), modified vaccinia virus Ankara (MVA85A), vaccination with HSP65 DNA (DNAhsp65), and Cytomegalovirus vector approaches such as RhCMV/TB (CMV-6Ag) and MTBVAC (344, 345). Conversely, for protein-adjuvant strategies like vaccine candidate ID93+GLA-SE, which contains Mtb antigens confirmed to contain immunodominant CD8+ T-cell epitopes (338), preclinical and clinical trial data to date suggest that a paucity of this subset is driven by this platform delivered intramuscularly (45, 46). Some strategies, like that for VMP1002, a recombinant BCG vaccine, are designed to drive CD8+ T-cell responses by enhancing vector apoptosis and autophagy, resulting in greater cytosolic antigen availability for MHCI presentation (29), which are only weakly induced in human infants by parental BCG vaccines (347, 348).

Along with platform of vaccination, route of delivery (also discussed further below) may play a critical role in eliciting pulmonary CD8+ T cells. In a mouse model, intranasal (i.n.) vaccination with an adenovirus vaccine drives mucosal CD8+ Mtb-antigen specific T cells that have distinct effector memory phenotypes and are maintained separate from circulating populations (325), suggesting that CD8+ T cells can be elicited at mucosal sites *via* vaccination and may persist. In the remarkable paper showing that 6 of 10 NHPs were protected from infection with Mtb in the cohort receiving i.v. BCG, CD8+ T cells were enriched in the airways compared to i.d. vaccinated groups (16). Importantly, vaccine-induced CD8+ T cells contribute to reducing bacterial burden in preclinical models of TB (325, 331, 345, 349). Our group has shown that a protein prime/Ad5 vector boost induces both CD4+ and CD8+ T cells and induces long-lived immunity in a preclinical mouse model of TB (350). Other groups have similarly demonstrated that this heterologous “prime-boost” strategy helps drive CD8+ T-cell responses *in vivo (* 350–355). With the advancement of novel and existing viral vectored and nucleic-acid-based vaccines, the focused targeting of Mtb-antigen-specific CD8+ T-cell responses is set to expand (344). We expect that the identification of high-priority immunodominant Mtb antigens containing CD8+ T-cell epitopes (337, 338, 356, 357) will further help drive candidate design for the heterologous immunity contributing to Mtb control.

### γδ T Cells

*Effector functions.* CD3+ T cells that express a unique combination of Vγ and Vδ in their TCR are known as γδ T cells. They depend heavily on junctional diversity, and in humans, there is an interesting preference for Vγ9Vδ2 pairs (358). They are less abundant than other T-cell types, making up 2%–10% of total circulating T cells in healthy individuals (359), and seem to fill a niche in peripheral tissues including mucosal airways, gastrointestinal tract, and epithelium (360). This mucosal preference makes them an enticing cell type to study for TB vaccine strategies. γδ T cells interact with many other cell subsets including DCs, NKs, and CD8+ T cells (359). Their location along with the ability to produce proinflammatory cytokines, including IL-17 and IFNγ, make them well suited to help in tissue repair and early recognition and combat of pathogens (360).

Unlike conventional αβ CD3+ T cells that recognize peptide antigens *via* MHC presentation, γδ T-cell subsets recognize non-peptide-based phosphorylated antigens and metabolic intermediate molecules with co-stimulation but without antigen processing or MHC presentation (361, 362). This categorical difference may help explain why γδ T cells are able to quickly respond to stimuli. However, in human age stratification studies, γδ T cells are able to form resident memory and persist in tissues after pathogen clearance (363), making them an interesting adaptive subset with early innate characteristics (361).

*Role in Mtb infections.* In preclinical models, γδ T cells have been shown to traffic to the airways and express IFNγ and IL-17 early after Mtb challenge (364). Indeed, early induction of IL-17, in particular, seems to be dominantly produced by γδ T cells and not CD4+ T cells, promoting early control of Mtb and subsequent granuloma formation (360, 361, 365). IL-17 is known to be protective against Mtb, as mice with depleted IL-17 are more susceptible to Mtb (366) and humans with specific IL-17 allelic genotypes are at a decreased risk of TB disease (367). There is an interesting dichotomy between the reduction in IL-17 production and an observed increase in T-cell exhaustion, including PD-1 expression, in individuals with TB disease, suggesting that beyond its pro-inflammatory role, IL-17 may also help regulate T-cell exhaustion profiles during disease states (368). In several clinical studies, adult and adolescent patients with active TB have been shown to harbor robustly higher cytolytic and IL-17-producing γδ T cells compared with healthy controls (369–371) (**Figure 2**), and this expansion decreases with TB drug treatment (362). Interestingly, some studies have reported a progression of TB disease severity to correspond with a decrease in circulating γδ T cells (372). Connecting these trends is difficult as TCR sequencing has revealed that distinct clones populate the periphery and lung (371), suggesting that there may be dedicated roles for local and disseminated control. Importantly, human Vγ9Vδ2 T cells are able to kill intracellular and extracellular Mtb *in vitro via* granulysin and perforin (373) and therefore may play both a direct and an indirect role in pathogen control.

*Vaccine induced.* Readouts for functional γδ T cells have largely been left out of many TB vaccine candidate assessments. Interestingly, while γδ T cells can respond to BCG immunization, depletion of this subset did not reduce protection from BCG challenge in a mouse model (371, 374). However, like CD8+ T cells, this may be an underappreciation of their full contribution to TB control. For instance, Vγ9Vδ2 T cells have been shown to provide help to CD8+ and CD4+ T cells (375), and this help may be largely unrecognized in preclinical models at singular time points. Indeed, rhesus macaques that received a mucosal immunization of vaccine candidate *L. monocytogenes* (Lm ΔactA prfA*) expressing Vγ2Vδ2 T-cell antigen (E)-4-hydroxy-3-methyl-but-2-enyl pyrophosphate (HMBPP) had a more rapid TH1 CD4+ and CD8+ T-cell response (376). These nonhuman primates also demonstrated reduced dissemination of Mtb post challenge compared to cohorts not immunized with the HMBPP vaccine candidate (376).

Early *in vitro* studies suggested that human monocytes infected with live Mtb were superior at activating and expanding Vγ2Vδ2 T cells compared to heat-killed Mtb preparations or protein antigens (377). These data may help explain why the majority of vaccine studies that note γδ T-cell contributions are replicating platforms. For example, a non-statistically significant increase in IL-17-producing γδ T cells was observed in cohorts of rhesus macaques immunized intradermally with MTBVAC and BCG compared to saline controls (24). In mouse models, it has been shown that γδ T cells are critical for vaccinia virus-induced CD8+ T-cell responses (378), which may have further implications for nucleic acid- or viral-based TB vaccine platforms (**Figure 1**). As tools for studying γδ T cells expand, the research community will better understand and dissect their possible role(s) in protective immunity.

## Review of Clinical Stage Vaccine Candidates and Strategies

Efficacious, durable, and globally available TB vaccines are on the horizon. The diverse representation of candidate platforms and antigen usage in the pipeline demonstrate how the research community is advancing despite the absence of robust continual funding needed. An update of completed and ongoing TB vaccine clinical trials is presented here (379). This is not a trivial endeavor as the Mtb genome encodes thousands of proteins, so protective antigen selection alone is a hurdle. For example, antigen selection and dose have been shown to directly influence T-cell differentiation and subsequent protection in therapeutic mouse models where too high antigen burden *in vivo* reduces vaccine efficacy and drives more terminal differentiation and less memory formation of T cells (380–382). When we examine clinical stage TB vaccine candidates (based on the Tuberculosis Vaccine Initiative Pipeline Tracker 2021) for preclinical or clinical evidence of the cell subsets reviewed above, it is evident that there is a paucity of surveillance for most innate subsets and a lack of uniformity for exploring adaptive responses (**Table 1**). Not surprisingly, most vaccine candidates that have evaluated a reported innate subset induction were subunit strategies that are affirming adjuvant properties. In **Table 1**, we included data generated from *in vivo* or *ex vivo* animal models or human clinical trial data that specifically identify subset-specific results. Some data that were excluded were T-cell ELISPOTs or proliferative assays, which did not specify a subset or phenotype assayed. Many of these subsets may fall into secondary endpoints for clinical studies but are not well followed up or reported if the findings are negative. Diversifying secondary immunogenicity endpoints and including early innate markers of vaccine-induced responses would only help the field continue to down-select those cell subsets or combinations that most significantly correlate with specific types of protection.

While **Table 1** captures an overview of cell subsets identified to be vaccine-induced in preclinical or clinical studies, there are particularly important nuances not detailed including the following: response kinetics, magnitude, lung homing, or memory and effector phenotypes, which are sure to influence protection. For example, route of vaccine delivery, may specifically skew mucosal immunity. The compartmentalization of immune responses between peripheral and local mucosa is well reviewed here (383). Recent studies have shown that aerosol and mucosal delivery of TB vaccine candidates help drive some IL-17-dependent cellular immunity (384) and increase vaccine-induced protective IgM antibody responses (385). Aerosol delivery of an adenovirus TB vaccine candidate has been described as safe and induces enhanced polyfunctional airway responses, including CD8+ T cells, outperforming intramuscular delivery of the same vaccine in mouse models of Mtb challenge (349, 386) and a phase 1 human clinical trial (387). A similar observation was made for an influenza vectored TB vaccine candidate in an i.n. administration to mice (388). Our own group has demonstrated that mucosal delivery of ID93+GLA-SE in mice converts the response from a TH1 CD4+ T-cell response typically seen following parenteral immunization, to a TH17 CD4+ T-cell response (52). The field has also demonstrated that i.n. BCG administration provides enhanced protection in mice and guinea pigs compared to subcutaneous delivery (389–391). Both the adaptive (392) and, most recently, trained innate immune responses (393) have been independently characterized for pulmonary BCG delivery-induced protection. Heat-killed MTBVAC delivered intranasally induces mucosal IgA, IgM, and IgG responses in both mouse and NHP models (315). In summary, mucosal delivery fundamentally changes the composition, localization, and functionality of vaccine-induced responses. In addition to inducing robust airway responses, mucosal delivery of TB vaccine candidates allow for dose sparing (394), which is an essential consideration for products intended for LMICs.

Selection of secondary immunogenicity endpoints and alignment across vaccine studies will likely vary slightly between POI and POD strategies. POD endpoints will likely more heavily rely on memory adaptive immunity whereas POI will be more innate and mucosal based. However, many candidates are being evaluated for both POI and POD indications (**Table 1**) and so comprehensive testing across the spectrum of cells discussed is likely warranted. Many other target population characteristics are also important to consider and may dramatically change the vaccine-induced immune response. TB vaccine candidates can be designed to be broadly effective across population heterogeneity or specific for different groups of people including naïve individuals or those who are BCG vaccinated, persons living with HIV, and individuals with latent TB, recovered from TB, or with TB disease recurrence (395). For example, more balanced CD4+ and CD8+ T-cell responses were observed in BCG primed individuals receiving Ad5Ag85A than BCG naïve individuals (32). Furthermore, the Ad5Ag85A candidate is likely best as a targeted childhood boost of BCG, as anti-Ad5 vector immunity increases with age and may reduce efficacy. “Prevention”, therefore, will have different definitions depending on the strategy as described above. Phase 3 clinical trials evaluating prevention are difficult as they require large numbers of participants followed for extended (2–5 years for POD) lengths of time. POI and POD vaccine strategies, target populations, efficacy endpoints, and novel strategies are outlined below.

POI target populations are largely pre-infected or naïve/unchallenged and a primary efficacy readout for these clinical studies would be remaining IGRA negative for a specific follow-up period. Prevention for POI in this case is absence of infection and likely a lack of measurable infection-mediated adaptive immune responses (e.g., IGRA-negative RSTRs). Populations with regular high exposures to Mtb, such as healthcare workers and household contacts of persons with active disease, are ideal for POI studies since they have a higher chance of becoming challenged than the general population, allowing for smaller trial numbers. For example, the recent phase 2b trial of ID93+GLA-SE completed in BCG-vaccinated healthcare workers in South Korea required just 107 participants to study two doses of vaccine against placebo (ClinicalTrials.gov Identifier: NCT03806686). These community participant trials are needed because there is currently no uniform human challenge model of Mtb, although robust advances in the last few years have made this more feasible. Experimental medicine studies in healthy volunteers leveraging BCG for pulmonary mycobacterial challenge may help serve as a bridge in the field and advances in this model are being made in Cape Town, South Africa (396, 397) as well as Oxford, UK (ClinicalTrials.gov Identifier: NCT02709278 and NCT03912207).

Conversely, POD strategies have a target population, which is post-Mtb challenge, and a primary efficacy readout is an absence of clinically active TB symptoms whereby the individual clears the infection (e.g., IGRA reversion) or remains latently infected for a period of follow-up. The complexities of the spectrum of latency and the research communities’ dated definitions of latency (harboring live bacteria that may reactivate versus a memory immune response to infection) and cure (clearance of bacteria) are recently discussed here (398). These definitions are not trivial as they are composed of information collected from clinical, pathological, microbial, and immunological sources but importantly inform vaccine trial endpoints (398). Better agreement in defining and testing for POD in latent individuals in particular would be beneficial for the field. Advances in host correlate of risk (COR) for progression to active disease signatures (399–402) are being evaluated as more short-term endpoints that can inform efficacy outcomes for drug and vaccine candidate regimens.

Exceptional efforts to mathematically model which vaccine strategies will most disrupt the global burden of TB disease have largely been led by the TB Modeling Group from the London School of Hygiene and Tropical Medicine. Most recently, the population-level impact of candidate POD or POI TB vaccines was modeled for China, South Africa, and India as representative high-burden regions with different demographics, healthcare infrastructures, and epidemiological trends. Interestingly, these models suggest that a candidate POD vaccine with ~70% efficacy for adolescents/adults already infected with Mtb would have the largest effect of incidence rate reduction (IRR) in all populations tested over a targeted 10-year mass vaccination campaign period (403), whereas the IRR was more variable by population for POI vaccine candidates depending on amount of transmission and less effective in regions modeled with reactivation as a primary driver (403). These findings do not seem unique to drug-sensitive Mtb, as this same Modeling Group next examined the influence of POI or POD vaccine candidates on emergence of DR in China and India as two representative regions that account for nearly 40% of the total global distribution of Mtb drug resistance (9). Interestingly, a vaccine with POI and POD efficacy had the largest influence of reducing DR incidence rates in both India and China (>70% in both models) with a predicted 2 million cases prevented in both regions (404), whereas a targeted vaccine with POI efficacy alone was nearly cut in half comparatively (404). Cost-effectiveness of these vaccine strategies against DR Mtb was dependent on regional wealth scenarios, efficacy, and POI or POD strategies, suggesting that a low-cost and age-targeted vaccine should still be a main priority for the pipeline (404, 405). Indeed, modeling deployment of M72/AS01_E_ for populations in South Africa and India suggests that this vaccine candidate would be cost-effective in all scenarios for South Africa based on thresholds for disability-adjusted life-years (DALY) avoided, but that adolescent vaccine campaigns in India would be most cost-effective (406).

## Animal Modeling for Vaccine Efficacy

The integration of animal models across the pipeline will only help to facilitate translational advancement of vaccine candidates. While each main model used (mouse, guinea pig, and NHP) has their own caveats for lack of fidelity to human infection, disease progression, and pathology, they do still make up a functional pipeline with tools for Mtb challenge, immunological study, and pathology interrogations. A summary of strengths and weaknesses of each model is presented here (407). Homogeneity of in-bred models is a common complaint or worry for preclinical modeling. However, the Collaborative Cross (CC) and Diversity Outbred (DO) mouse models are elegant systems to examine host–pathogen interactions and uncover more detailed mechanisms of vaccine-mediated protection (408), including those in TB (409, 410), and we expect these genotypes to be further leveraged to examine the contribution of cellular subsets to vaccine-mediated immunity in the near future. These detailed studies should carefully profile and capture the kinetics of cell activation, metabolic states, trafficking, and contribution to containment in early, middle, and late time points post challenge. The ultra-low dose challenge mouse infection (1–3 CFU per mouse), well defined and leveraged by the Urdahl research team, is another recent advancement bringing greater harmony across models that are more physiologically relevant and representative of human infections with resulting heterogeneous acute bacterial loads (411). Historically, the mouse model represents acute and chronic TB disease, but offered little symmetry with human latent TB immunology and protection against reinfection. Recently, researchers have pioneered a small animal model of “contained Mtb infection” (CMTB). In this model, live Mtb is administered into a mouse ear where it persists, which will be exceedingly useful for differentiating immune profiles of active and latent disease and for evaluating protective responses (412, 413). The next model in the three-tiered pipeline is commonly rabbits or guinea pigs, which are readily infected with aerosolized Mtb and demonstrate more robust pathology, including human-like necrotic granulomas, than mice. A lack of immunological resources makes these models less than ideal for evaluating immune mechanism(s) of protection; however, they are a readily available model for vaccine and drug safety (PPD and DTH responses) and efficacy evaluations (414). With the more widespread adoption of *in situ* transcriptional evaluations and pioneering researchers generating more immunological tools, the guinea pig will likely become more beneficial for COP interrogations in the coming decade. NHPs are the final model in the preclinical pipeline, which demonstrate the closest alignment with human TB disease and immunology. These surrogates are used for a wide array of human diseases and interventional studies, which can significantly hamper their availability, as experienced during the SARS-CoV-2 pandemic (415). NHPs serve as not only an ideal model for late-stage vaccine candidates but also a valuable tool for dissecting specific immune COP, especially in light of the best protection data in NHPs observed to date with the administration of i.v. BCG (16). AS surrogate endpoints become more predictive of efficacy for human protection, with or without reliance on mechanism, we expect them to be evaluated and even back translated through the pipeline. An example are host risk signatures predicting progression to active disease, which were identified in human clinical cohorts (416–421) and evaluated in preclinical models (422).

## Discussion

This review is not exhaustive of all cellular compartments or agents and many subsets have been well reviewed elsewhere. Indeed, many of these cells or antimicrobial mediators may play significant roles in the prevention of Mtb infection, as signs of measurable memory immune responses in some cases are not evident, and could explain why some individuals are resistant to infection. For example, while trained innate immunity has been widely studied in the HIV research community, it is more recently being comprehensively followed by the TB research community (423). Trained innate immune cells, including myeloid lineages and NK cells, are those that display a “memory-like” phenotype with metabolic and epigenetic reprogramming (164, 423, 424). This strategy is of particular interest for live-attenuated vaccine strategies like BCG and BCG-based candidates and strategies with adjuvants that differentiate innate cells through PRR pathways and induce specific metabolic phenotypes (425). Donor-unrestricted T cells (DURTs), including γδ T-cell subsets discussed above, are exceedingly unique in that they are not restricted by MHC presentation of antigen but rather recognize non-peptide antigens on unique non-polymorphic molecules including Cluster of Differentiation 1 (CD1), MHC-related 1 (MR1 molecules), HLA^E^, and butyrophilin 3A1 (296, 426). These non-MHC molecules are genetically shared across heterogenous populations and so the responding cells are not donor-restricted in the way classical TCRs are, making them attractive vaccine targets as they may be more widely immunogenic across diverse regions. Generating tools to better study DURTs are a significant research priority for the field, including generation of DURT antigens and amenable vaccine formulations (426). Indeed, the non-peptide nature of DURT antigens makes them candidates for incorporation into formulations such as emulsions and nanoparticles, which could further be complexed with classical vaccine T-cell antigens as a novel vaccine strategy. MAIT cells are T cells that are similarly non-classically restricted but with a propensity for mucosal homing, recognizing antigens presented on MR1 with rapid response capabilities and the functional capacity to kill infected cells (296, 427, 428). However, their active role is somewhat confusing as some clinical data demonstrate that individuals with TB have decreased numbers of circulating MAIT cells with reduced activation (429), whereas others are detected with tetramers at similar frequencies in peripheral blood from TB-infected and uninfected individuals (427). In mouse models of TB challenge, MAIT cells have been shown to delay T-cell priming early during challenge but are efficient targets of host-directed therapies (428), making them an interesting target in vaccine and drug therapy strategies. Beyond cell subsets, antimicrobial peptides (AMP) likely play a significant role in POI mechanisms. AMP are a wide array of multi-functional peptides secreted by innate immune cells. They are capable of targeting bacteria, viruses, and fungi. AMPs are broadly classified by their structural characteristics and amino acid composition, but all share similar mechanisms for targeting pathogens. AMPs are known to primarily target the pathogen membrane through permeation, although there has been evidence of AMPs also disrupting DNA/RNA synthesis and inducing ATP loss (430). AMPs identified in the human defense against mycobacteria include cathelicidin, defensins, hepcidin, lactoferrin, azurocidin, elastases, antimicrobial RNases, eosinophil peroxidase, cathepsins, granulysin, and lipocalin2 (430). While these AMPs are derived from many different types of cells, the overwhelming majority are innate and likely play early roles in protection from mycobacteria and may contribute to RSTR phenotypes. More work needs to be done to determine if genetics plays a role in abundance or efficacy of AMPs against Mtb.

While many vaccine strategies are benchmarked against induction of robust Mtb-antigen-specific CD4+ TH1 responses, the data reviewed here and elsewhere make a strong case for inclusion of many other subsets depending on POI or POD strategy. While heterologous vaccine strategies such as subunit + adjuvant prime with vector-based candidate boost may better drive multifaceted adaptive immunity, their clinical deployment may be further delayed than present candidates. However, developing and screening vaccine platforms that induce specific combinations of immunity against high-priority Mtb protective antigens should be a major strategy for the pipeline (**Table 2**). While it is true that candidates that show efficacy in the preclinical pipeline may not ultimately be successful in clinical trials (20), if we begin to look across candidates and compile more data, trends that better predict success may emerge. When we evaluate the many cell subsets that contribute to Mtb control, it is essential to consider (1) the time in which they contribute, (2) the way they interact with other cell subsets, and (3) how they may be preferentially induced by a vaccine regimen platform, route of delivery, and dosing interval (**Table 1**). As a research community, we need better immune correlates that are mechanistic or that better predict clinical efficacy. The simultaneous scrutiny of vaccines and their potential for public health impact on a global scale have never been higher. As national and international consortiums of TB vaccine developers and researchers align across the pipeline, we may soon have reliable models and vaccine endpoints that more readily steer candidates to approval and clinical deployment, relegating TB disease to a thing of the past.

**Table 2:** Markers of major cell subsets.

Cellular Subset	Mouse Markers	Human Markers
Neutrophils	CD11b^+^, Ly6G^+^, Ly6B^-^	CD15^+^, CD16^+^
Alveolar Macrophages	CD11b^lo^, CD11c^+^, Siglec F^+^	CD206^+^, CD163^+^, CD169^+^, HLADR^+^
Dendritic Cells	CD11c^+^, MHCII^+^	CD1c^+^, CD83^+^, CD141^+^, CD209^+^, MHCII^+^
Natural Killer Cells	CD3^-^, NK1.1^+^, CD56^+^	CD3^+^, CD16^+^, CD56^+^
B Cells	CD19^+^, IgM^±^	CD19^+^, IgM^±^
CD8^+^ T Cells	CD3^+^ CD8^+^ CD107a^±^	CD3^+^ CD8^+^ CD107a^±^
γδ T Cells	CD3^+^ Panγδ^+^ CD161^+^	CD3^+^ Panγδ^+^

## Author Contributions

All authors (SL, DW, MR, RC, and SB) helped conceptualize the manuscript. SL, DW, MR, and SB drafted the manuscript. SL, DW, and MR drafted figures and tables. All authors (SL, DW, MR, RC, and SB) reviewed the manuscript and contributed to edits. All authors contributed to the article and approved the submitted version.

## Funding

This review was supported by the National Institute of Allergy and Infectious Diseases (NIAID) of the National Institutes of Health (NIH) under award number R01AI125160. BDW was supported through the University of Washington, Diseases of Public Health Importance training grant number T32AI007509.. The content is solely the responsibility of the authors and does not necessarily represent the official views of the National Institutes of Health.

## Conflict of Interest

The authors declare that the research was conducted in the absence of any commercial or financial relationships that could be construed as a potential conflict of interest.

## Publisher’s Note

All claims expressed in this article are solely those of the authors and do not necessarily represent those of their affiliated organizations, or those of the publisher, the editors and the reviewers. Any product that may be evaluated in this article, or claim that may be made by its manufacturer, is not guaranteed or endorsed by the publisher.
